# Systematic Model Peptide Studies: A Crucial Step To Understand the Coordination Chemistry of Mn(II) and Fe(II) in Proteins

**DOI:** 10.1021/acs.inorgchem.4c05380

**Published:** 2025-03-11

**Authors:** Karolina Pawlik, Malgorzata Ostrowska, Elzbieta Gumienna-Kontecka

**Affiliations:** Faculty of Chemistry, University of Wrocław, Wrocław 50-383, Poland

## Abstract

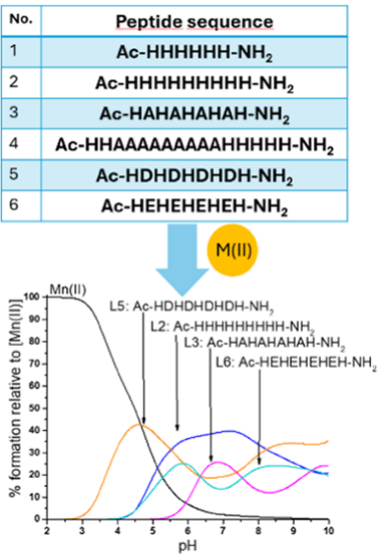

Pathogenic bacteria and all other species require Mn(II) and Fe(II) ions for proper growth. Microbes use a variety of assimilation pathways to obtain the necessary metal ions, and their metal homeostasis mechanisms are still not fully uncovered. The knowledge of the poorly discovered complexation chemistry of Mn(II) and Fe(II) ions could help us to understand the basis of those processes better. We have designed six model peptides (L1 – Ac-HHHHHH-NH2, L2 – Ac-HHHHHHHHH-NH_2_, L3 – Ac-HAHAHAHAH-NH_2_, L4 – Ac-HHAAAAAAAAAHHHH-NH_2_, L5 – Ac-HDHDHDHDH-NH_2_, and L6 – Ac-HEHEHEHEH-NH_2_) inspired by Mn(II) and Fe(II) binding motifs that are prevalent in nature, in order to clarify their coordination preferences. Spectrometric, spectroscopic, and potentiometric techniques were used to determine the thermodynamic and structural properties of the studied systems. All of the investigated ligands possess efficient Mn(II), Fe(II), and Zn(II) binding sites. Complex stability and metal affinity are significantly influenced by the length of the peptide sequences, as well as the location and quantity of coordinating amino acid residues like His, Asp, and Glu.

## Abstract 2

Metal ions are essential for all living organisms, in part due to their interactions with proteins. Mn(II) and Fe(II) are required by pathogenic bacteria for proper growth and establishing full virulence; however, their assimilation pathways and coordination chemistry are still very poorly understood. We have designed 6 model peptides based on the binding motifs commonly found in proteins, and in this work, we characterize their coordination chemistry with Mn(II), Fe(II), and Zn(II) ions.

## Introduction

Metal ions are deemed essential for all living organisms because of their crucial roles in various biological systems. One of the key aspects of the need for metal ions lies in their interactions with proteins, where they often serve as cofactors, regulators, or perform structural functions.^1−3^ Metal ion discrimination in biological systems is generally thought to involve an interplay between the prevailing metal ion availability, its chemical properties, the nature of coordinating ligands, geometric preferences, and finally, the effect of the Irving-Williams stability series (where affinity, in order of increasing atomic number, can be represented as Mg(II)/Ca(II)<Mn(II)<Fe(II)<Co(II)<Ni(II)<Cu(II)>Zn(II) with “>” indicating higher affinity) on protein-metal ion affinity.^4−6^ These factors also influence the biological functions performed by metal ions. Despite the fact that, from the perspective of fundamental inorganic chemistry, it is true that Mn(II) and Fe(II) complexes tend to be far less thermodynamically stable than other biologically important first-row transition metals, they are still among the metal ions playing a crucial role in innumerable biochemical reactions of all living organisms, especially in pathogens and in the host immune system.^7−9^ The decreased thermodynamic stability may act to the advantage of the machinery involved in maintaining metal ion homeostasis, by aiding kinetically accessible transport.^7^ The basis for understanding these processes is the insight into the coordination structures and binding affinity of the key players involved in metal ion homeostasis.

As the concentration of essential metal ions should be kept in optimal ranges to maintain proper functionality, organisms have evolved tightly regulated systems to preserve homeostasis.^9−11^ Focusing on homeostasis at the host–pathogen interface, to ensure maximum proliferation and full virulence during an infection, the bacteria aim to gain all essential nutrients from the host. Pathogens utilize a broad range of approaches, one of them being high-affinity metal ion importers. These importers are rarely selective and mostly transport a broad range of essential transition metal ions.^7,12−14^ The host organism also employs various strategies to compete for essential nutrients with invading microbes and sequester them from the invaders. One of these strategies is nutritional immunity—a strategy involving an impoverishment of essential metal ions at infection sites using specialized proteins.^8,9,12,15^ The first step to understanding how these systems are able to select appropriate metals over others is an insight into their coordination chemistry to elucidate different binding sites, thermodynamic features, and structural details. The main constraint on the way to understanding Mn(II) and Fe(II) homeostasis is a negligible number of papers concerning the coordination, structure, stability, and mode of action of Mn(II)–peptide complexes. In contrast to other biologically important transition metal ions, for which one can find a broad data library, in the case of Mn(II) and Fe(II), to the best of our knowledge, literature data are limited to a strikingly small number of papers, underlying the need for further systematic studies. To fill this gap, we have decided to perform systematic studies on the coordination properties of carefully designed model peptides toward Mn(II) and Fe(II) ions.

Proteins, crucial for metal transport and homeostasis, both for the host and pathogens alike, are often rich in histidine (His), along with aspartic acid (Asp) and glutamic acid (Glu) residues. These essential amino acids can very efficiently bind a variety of metal ions.^16−19^ The position and number of His residues have a vital influence on coordination mode, thermodynamic stability, and mode of action, and thus are considered important factors in the determination of the thermodynamic properties of transition metal complexes with His-rich proteins and model peptides.^16,18^ Small model peptides, rich in metal-binding amino acids, can mimic the binding sites of various biologically relevant proteins. Studies of these peptides with transition metal ions could help us understand the factors that drive the complexation process, especially in the case of metals with poorly known coordination chemistry in relation to peptide complexes such as Mn(II) and Fe(II) ions.

To study the influence of the number, position, and occurrence of amino acids with high affinity to transition metal ions (His, Asp, and Glu), we have designed six model peptides (Table 1) mimicking simplified, naturally occurring metal ion binding sites in proteins. Three metal ions were selected for this study: Mn(II), Fe(II), and Zn(II) ions. Both Mn(II) and Fe(II) ions have similar coordination preferences and can occupy the same binding sites, as evident in broad-specificity bacterial metal ion transporters.^7,12,13^ However, the coordination chemistry of these ions, including peptide ligands, is poorly known, which is why in this work we focus on exploring the interactions between Mn(II) and Fe(II) ions with selected model peptides containing His, Glu, and Asp residues (Table 1). Zn(II) ions are one of the most prevalent trace elements in the human body, often demonstrating a high affinity toward Mn(II)-binding proteins. In addition, there is also some evidence that Fe(II) and Zn(II) can influence Mn(II) acquisition.^20−22^ For Zn(II) ions, one can find a broad data library concerning solution studies with peptide ligands rich in His, Glu, and Asp residues. Despite this, we have decided to expand the knowledge and complete our investigations on Zn(II) ions to have a direct comparison of the data obtained for Mn(II) and Fe(II) ions for all studied systems. Spectroscopic and potentiometric techniques were used to determine the thermodynamic and structural properties of the studied systems.

**Table 1:** Sequences of Studied His-Rich Model Peptides

name	model peptide sequence
L1	Ac-HHHHHH-NH_2_
L2	Ac-HHHHHHHHH-NH_2_
L3	Ac-HAHAHAHAH-NH_2_
L4	Ac-HHAAAAAAAAAHHHH-NH_2_
L5	Ac-HDHDHDHDH-NH_2_
L6	Ac-HEHEHEHEH-NH_2_

## Experimental Section

### Materials

All investigated ligands were purchased from KareBay and Biochem and were of 98% purity. The identity of the peptides was confirmed by mass spectrometry. The purity of the used ligand was determined by potentiometric titrations using the Gran method.^23^ Carbonate-free 0.1 M Titripur sodium hydroxide was purchased from Sigma-Aldrich and standardized by potentiometric titration with potassium hydrogen phthalate (Sigma-Aldrich). All of the solutions used were prepared using double-distilled water. The ionic strength of all samples was adjusted to *I* = 0.1 M by the addition of sodium perchlorate (Sigma-Aldrich). Ligand samples also contained 4 mM perchloric acid (J.T. Baker). Solutions containing Zn(II) and Mn(II) ions were made from corresponding metal perchlorate salts (Sigma-Aldrich) and standardized using two independent methods: inductively coupled plasma optical emission spectrometry (ICP-OES) and complexometric titrations with standardized ethylenediaminetetraacetic acid disodium salt (Na_2_H_2_EDTA) and murexide. Solutions containing Fe(II) ions were prepared and standardized under an inert atmosphere immediately before the experiments. The solutions were prepared using ammonium iron(II) sulfate (Sigma-Aldrich) and standardized using a 1,10-phenanthroline (Sigma-Aldrich) colorimetric assay. As a consequence of the high oxidation susceptibility of Fe(II) ions, all experiments were performed in an argon atmosphere inside a glovebox using deoxygenated solvents. In mass spectrometry experiments, no peaks assigned to Fe(III) complexes were observed. In solutions, Mn(II) ions are not susceptible to aerial oxidation in both acidic and neutral pH. In alkaline solutions, insoluble Mn(II) hydroxides (Mn(OH)_2_) precipitate. Those hydroxides are susceptible to aerial oxidation. In a few of our potentiometric experiments, especially regarding weaker Mn(II) complexes, we have observed precipitation of white sediment at alkaline pH (∼10). The precipitate turned dark, brownish color sometime after opening the potentiometric cell, proving to be Mn(OH)_2_, and oxidizing to MnO_2_ due to contact with oxygen, as seen in Figure S1. Moreover, we have not observed any peaks associated with higher oxidation states of manganese on ESI-MS or EPR spectra.

### Electrospray Ionization Mass Spectroscopy (ESI-MS)

ESI-MS experiments were performed using a Bruker Q-TOF compact mass spectrometer. Spectra were measured in positive-ion mode and contained a 0.1 mM concentration of the ligand. Each sample was prepared in a 50:50 (v/v) methanol/water mixture at pH 3, 6, and 9 with 1:1, 1:2, 2:1, and 3:1 metal/ligand molar ratios. The TuneMix mixture (Bruker Daltonics) was used to calibrate the instrument. The instrumental parameters were: scan range *m*/*z* = 100–1900; dry gas, nitrogen; *T* = 170 °C; capillary voltage, 4500 V; ion energy, 5 eV. The samples were infused at a flow rate of 3 μL/min. The data were processed with the use of the Compass Data Analysis 4.0 software (Bruker Daltonics). All of the solvents used were of liquid chromatography–mass spectrometry grade.

### Potentiometric Titrations

Potentiometric titrations were performed using a Metrohm Titrando 905 titrator connected to the Dosino 800 dosing system and a Metrohm OMNIS titrator connected to the OMNIS dosing module. The pH of a solution was measured by a pH electrode, an InLab Semi-Micro (Mettler-Toledo) for the Titrando 905 titrator, and a Biotrode Semi-Micro electrode (Metrohm) for the OMNIS titrator. The glass cell used for measurements was equipped with a microstirrer, a microburet delivery tube, and an inlet–outlet tube for argon. The electrode was calibrated every day for hydrogen ion concentration by performing a titration of 3 mL of 4 mM perchloric acid solution with sodium hydroxide. The electrode characteristics, such as standard potential and the slope, were computed by means of the GLEE program.^23^ The tested solutions contained 0.5 mM ligand concentrations, 4 mM perchloric acid concentrations, and 0.1 M sodium perchlorate concentrations (ionic strength). The measurements were performed at a 1:1.1 metal/ligand molar ratio. The precise concentrations of ligand solutions were determined using the Gran method.^24^ All titrations were performed under an argon atmosphere using a standardized carbonate-free solution of sodium hydroxide as a titrant. All stability constants of proton and metal–ligand complexes were calculated using titration curves obtained at 298 K and in the 2–11 pH range using *HYPERQUAD 2008*(25) and *SUPERQUAD*(26) software. The competition and speciation diagrams were created by using HYSS^27^ and OriginLab 2016 software. The Mn(II), Fe(II), and Zn(II) hydrolysis constants were taken into consideration during stability constant calculations of metal–ligand complexes. The constants at zero ionic strength were obtained from “The Hydrolysis of Metal Cations” by Brown and Ekberg^28^ and calculated to 0.1 M ionic strength with the formula proposed by Baes and Mesmer (Table S1).^29^

### Electron Paramagnetic Resonance (EPR) Spectroscopy

EPR spectra were recorded using a Bruker ELEXSYS E500 CW-EPR spectrometer equipped with an NMR teslameter (ER 036TM) and a frequency counter (E 41 FC) at X-band frequency at room temperature (298 K). The ligand concentration was 0.5 mM, and the metal/ligand molar ratio was 1:1.1. EPR parameters were obtained by using the Doublet New (EPR OF; S = 1/2) program by A. Ozarowski (National High Magnetic Field Laboratory, University of Florida, Gainesville, FL).

## Results and Discussion

### Ligand Protonation Constants

The dissociation constants of the studied free ligands and the stability constants of ligand–metal complexes were calculated using data obtained by potentiometric titrations, as presented in Table 2. All studied peptides were protected at both the N- and C-terminus by acetylation and amidation, respectively.

**Table 2:** Protonation Constants (log*β*^*H*^) and p*K*_a_ Values of the L1–L6 Ligands^a^

	ligands					
assignments	L1	L2	L3	L4	L5	L6
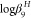		53.93(2)			47.00(2)	49.22(3)
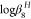		49.32(3)			44.78(2)	46.25(3)
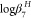		44.39(4)			41.79(1)	42.51(3)
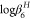	36.98(2)	38.93(6)		37.62(4)	38.22(1)	38.43(3)
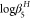	32.03(2)	33.35(6)	31.52(2)	32.42(3)	34.15(1)	33.76(3)
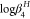	26.41(3)	27.36(6)	25.98(2)	26.66(5)	28.39(1)	27.04(3)
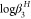	20.54(3)	21.09(5)	20.27(3)	20.7(4)	22.06(1)	21.61(4)
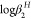	14.11(2)	14.53(2)	13.87(2)	14.14(4)	15.16(1)	14.86(2)
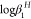	7.39(3)	7.42(3)	7.22(3)	7.48(5)	7.98(1)	7.74(3)
p*K*_*a*_		4.61 (H)			2.22 (D)	2.97 (E)
p*K*_*a*_		4.93 (H)			2.99 (D)	3.74 (E)
p*K*_*a*_		5.46 (H)			3.57 (D)	4.08 (E)
p*K*_*a*_	4.95 (H)	5.58 (H)		5.20 (H)	4.07 (D)	4.67 (E)
p*K*_*a*_	5.62 (H)	5.99 (H)	5.54 (H)	5.76 (H)	5.76 (H)	5.82 (H)
p*K*_*a*_	5.87 (H)	6.27 (H)	5.71 (H)	5.96 (H)	6.33 (H)	6.33 (H)
p*K*_*a*_	6.43 (H)	6.56 (H)	6.40 (H)	6.56 (H)	6.90 (H)	6.75 (H)
p*K*_*a*_	6.72 (H)	7.11 (H)	6.65 (H)	6.66 (H)	7.18 (H)	7.12 (H)
p*K*_*a*_	7.39 (H)	7.42 (H)	7.22 (H)	7.48 (H)	7.98 (H)	7.74 (H)

a *T* = 298 K, *I* = 0.1 M NaClO_4_, standard deviations on the last digit given in parentheses. Overall stability constants (β) expressed by the equation: β(H_*n*_L) = [H_*n*_L]/[L][H^+^]^*n*^. Acid dissociation constants (p*K*_a_) expressed as p*K*_a_ = logβ(H_*n*_L) – logβ(H_*n*–1_L).

L1 – Ac-HHHHHH-NH_2_ has 6 deprotonation constants (p*K*_a_), and thus in its fully protonated form can be described as [H_6_L]^6+^ ligand. L2 – Ac-HHHHHHHHH-NH_2_ has nine deprotonation constants and therefore can be considered an [H_9_L]^9+^ ligand. L3 – Ac-HAHAHAHAH-NH_2_ consists of five histidine (His) residues, each one separated by an alanine (Ala) residue, and can be described as [H_5_L]^5+^. L4 – Ac-HHAAAAAAAAAHHHH-NH_2_ ([H_6_L]^6+^) consists of six His residues, separated by a long (nine residues) poly-Ala chain. Both L5 – Ac-HDHDHDHDH-NH_2_ and L6 – Ac-HEHEHEHEH-NH_2_ behave like [H_9_L]^5+^ ligands.

All of the obtained dissociation constants for L1–L4 correspond to His side chain deprotonations. For L5 and L6, the first four deprotonation events can be assigned to aspartic acid (Asp) and glutamic acid (Glu) residues, respectively, while the next 5 constants can be attributed to His imidazole group deprotonations. The exact p*K*_a_ values of all ligands are listed in Table 2, and speciation diagrams are presented in Figure S2. The p*K*_a_ values of the carboxylic side chain groups lie in the range of 2.22–4.07 and 2.97–4.67 for Asp and Glu residues, respectively. The deprotonation of the imidazole groups of His occurs in the range of 4.61–7.98, depending on the ligand. The deprotonation constants remain in good agreement with those found in the literature for similar poly-His, poly-Asp, and poly-Glu systems.^30−36^

Due to the presence of numerous positively charged His residues in all studied peptides, in the acidic pH, we observe the formation of highly charged species, corresponding to fully protonated ligands (*vide supra*). This is the reason for the increasing acidity of most present amino acid side chains compared to p*K*_a_ values observed for free amino acids (p*K*_a_ = 3.86 for Asp, 4.07 for Glu, and 6.07 for His).^37^ Highly charged species become less stable; thus, they tend to aid deprotonation to increase system stability. With progressive deprotonation, the charge of the species decreases, causing an increase in the p*K*_a_ values of subsequent amino acid residues.^37,38^

### Metal Complex Stoichiometry

The stoichiometry of all formed metal complexes with model peptides was determined using ESI-MS experiments with 1:1, 1:2, 2:1, and 3:1 metal/ligand molar ratios. The experiments in the excess of metal ions were conducted for all metal ions, and at different pH (3, 6, 9), adjusted by the addition of 0.1 M HCl or 0.1 M NH_4_OH. We identified the presence of [Mn_2_L]^2+^ polynuclear complex in the experiment with the excess of 3:1 (metal/ligand) Mn(II) ions (see exemplary mass spectra in Figure S3). Besides that spectrum, only mononuclear 1:1 (metal/ligand) complexes are present under the experimental conditions employed. We identified no bis-complexes in our study. The exemplary mass spectrum for the 1:1 (metal/ligand) Mn(II)/L2 system is shown in Figure S4, as a representation of all measured spectra. The correct peak assignment was confirmed by comparisons of simulated and experimental isotopic distributions, which show excellent agreement. A comparison of the experimental and simulated *m*/*z* values of the signals of ionized ligands and metal complexes, present in the spectra of each metal/peptide system, is presented in Table S2.

### Metal Complex Thermodynamic Stability and Structure

#### Mn(II) Complexes

**Figure 1:**
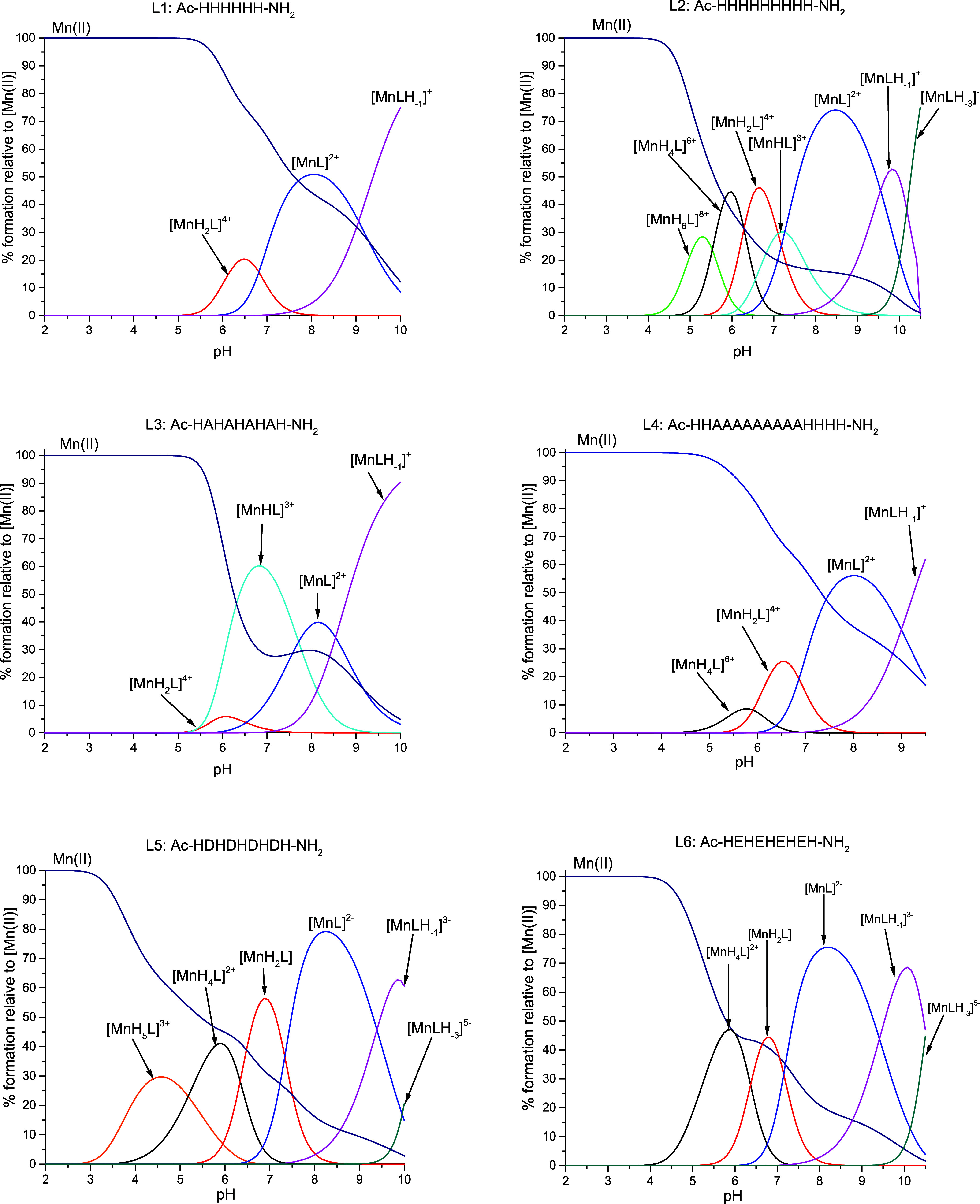
Species distribution diagrams of complexes formed between the Mn(II) and L1–L6 systems. Species distribution calculated for potentiometric titration experimental conditions. [Mn(II)_tot_] = 0.5 mM; M:L = 1:1.1.

For all ligands, calculations based on potentiometric data suggest the formation of mononuclear species with various protonation states (Table 3 and Figure 1).

**Table 3:** Stability Constants (log*β*), and p*K*_*a*_ Values of All Mn(II)/peptide Systems^a^

	ligands					
assignments	L1	L2	L3	L4	L5	L6
log*β* [MnH_6_L]		42.70(3)				
log*β* [MnH_5_L]					37.41(7)	
log*β* [MnH_4_L]		31.64(2)		29.53(6)	32.23(6)	31.68(8)
log*β* [MnH_3_L]						
log*β* [MnH_2_L]	17.43(4)	19.70(2)	17.03(6)	17.72(2)	19.55(5)	18.95(9)
log*β* [MnHL]		11.97(4)	11.68(5)			
log*β* [MnL]	3.80(3)	4.71(3)	3.97(4)	3.99(2)	4.88(5)	4.64(7)
log*β* [MnLH_–1_]	–5.27(2)	–4.88(4)	–4.57(5)	–5.01(6)	–4.51(5)	–4.77(9)
log*β* [MnLH_–2_]						
log*β* [MnLH_–3_]		–25.30(3)			–24.98(5)	–25.79(8)
p*K*_*a*_ [MnH_6_L]						
p*K*_*a*_ [MnH_5_L]						
p*K*_*a*_ [MnH_4_L]					5.07	
p*K*_*a*_ [MnH_3_L]						
p*K*_*a*_ [MnH_2_L]						
p*K*_*a*_ [MnHL]		7.10	5.35			
p*K*_*a*_ [MnL]		7.26	7.71			
p*K*_*a*_ [MnLH_–1_]	9.07	9.59	8.54	9.00	9.39	9.41
p*K*_*a*_ [MnLH_–2_]						
p*K*_*a*_ [MnLH_–3_]						

a *T* = 298 K, *I* = 0.1 M NaClO_4_, standard deviations on the last digit given in parentheses. Overall stability constants (β) expressed by the equation: β([MnH_*n*_L]^(*n*+2)+^) = [[MnH_*n*_L]^(*n*+2)+^]/[Mn(II)][[L]^*n*+^][H^+^]^*n*^. Acid dissociation constants (p*K*_a_) expressed as p*K*_a_ = log β ([MnH_*n*_L]^(*n*+2)+^) – log β ([MnH_*n*–1_L]^(*n*+1)+^).

For L1–L4 ligands, in which only His residues deprotonate, complexation starts around pH 4–5 with the first species containing a different number of deprotonated imidazole nitrogen atoms, depending on the ligands (four deprotonated His in L1, three in both L2 and L3, and two in the L4 system) (Table 3 and Figure 1). Subsequent complex forms, up to [MnL]^2+^, arise because of the deprotonation of the remaining His residues. The last species, [MnLH_–1_]^+^ and [MnLH_–3_]^−^, are probably the effects of water molecule deprotonation, as there is no evidence of Mn(II) ions engaging in amide nitrogen binding in peptide ligands. The presence of water molecules in the Mn(II) coordination sphere is common and occurs also in the binding sites of various metalloproteins.^39^

As most of the stepwise deprotonations of the Mn(II)/L1–L4 complex species are not observed, we cannot be sure about the number of His residues involved in the coordination of Mn(II) ions in specific complex species. Considering the literature data concerning the binding mode and thermodynamic characteristics of divalent metal ion complexes with ligands containing poly-His motifs, we can hypothesize the binding of Mn(II) ions by two or three imidazole groups, separated by nonbinding residues.^30,32,40^ It strongly suggests the coexistence of several complex forms in equilibrium, with different sets of imidazole nitrogen atoms binding to the central Mn(II) ions in each species. The phenomenon is known as “so-called” polymorphic binding sites.^30,35,41^ In addition, taking into account the direct proximity of His residues and the relative shortness of the used peptides, we can suppose that steric hindrance will prevent the simultaneous binding of all deprotonated residues.

A slightly different behavior is observed for L5 and L6 ligands as they contain Asp and Glu, respectively, next to His residues. For L5, the complexation process begins at a pH around 3, with the formation of species in which four Asp residues are deprotonated. For L6, the first species, in which four Glu and one His residues are deprotonated, forms at a higher pH around 4 (Table 3 and Figure 1). It is consistent with the lower acidity of Glu compared to that of Asp. The formation of the following species is derived from the deprotonation of imidazole nitrogen atoms, similar to previously described ligands. The subsequent deprotonation of three water molecules leads to the formation of [MnLH_–1_]^+^ and [MnLH_–3_]^−^ species. The deprotonation pattern is consistent with the previously described polymorphic binding site phenomenon, suggesting the coexistence of various complex forms with different sets of imidazole nitrogen donors.^30,35,41^

To confirm the formation of Mn(II)-peptide complexes, we have conducted room-temperature (RT, 298 K) EPR experiments in a broad pH range (Figures S5–S10).^42^ Mn(II) is a high-spin paramagnetic metal ion with a 3*d*^5^ valence electron configuration. In high-spin Mn(II) systems, the spin state is S = ^5^/_2_, and the nuclear spin state is I = ^5^/_2._ For high-spin Mn(II) complexes, we observe a very small anisotropy of the Zeeman interactions, which leads to a small *g* value, similar to that of a free electron (*g* = 2.0023). This behavior is typical for S-state ions with electron-density geometry close to spherical.

At RT EPR, the [Mn(H_2_O)_6_]^2+^ complex exhibits a distinctive X-band six-line pattern, centered at *g* = 2.002, resulting from hyperfine splitting of the allowed EPR transition. When water molecule ligands are displaced by the peptide amino acid side chains, the signal disappears (Figures S5–S10). Peptide-bound Mn(II) ions become EPR silent at room temperature because of the signal broadening beyond detection. The signal broadening is caused by zero-field splitting arising from the disturbance of the ligand field of Mn(II) ions. Thus, the signal observed in the RT EPR spectra comes only from unbound Mn(II) ions. The loss of the signal intensity, with the rise of pH, signifies Mn(II) ion binding to the L1–L6 peptides.^43,44^

#### Fe(II) Complexes

**Figure 2:**
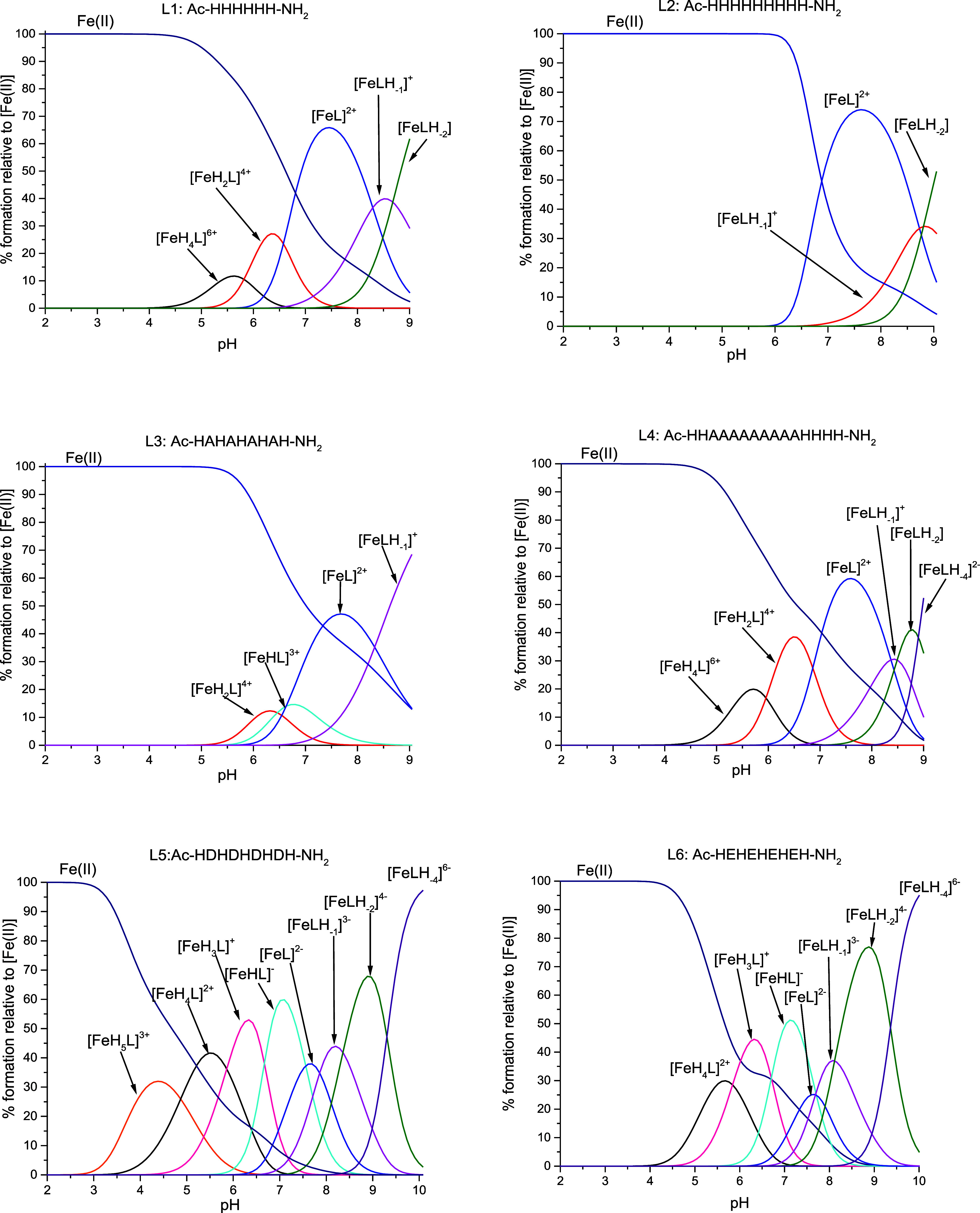
Species distribution diagrams of complexes formed between Fe(II) and the L1–L6 systems. Species distribution calculated for potentiometric titration experimental conditions. [Fe(II)]_tot_ = 0.5 mM; M/L = 1:1.1.

For all ligands, calculations based on potentiometric data suggest the formation of mononuclear species with various protonation states (Table 4 and Figure 2).

**Table 4:** Stability Constants (log*β*), and p*K*_*a*_ Values of All Fe(II)/Peptide Systems^a^

	ligands					
assignments	L1	L2	L3	L4	L5	L6
log*β* [FeH_6_L]		42.53(1)				
log*β* [FeH_5_L]					37.63(6)	
log*β* [FeH_4_L]	29.40(5)	30.90(2)		30.01(7)	32.76(7)	31.60(9)
log*β* [FeH_3_L]					26.90(6)	25.76(5)
log*β* [FeH_2_L]	17.90(2)	18.57(1)	17.01(8)	18.10(4)		
log*β* [FeHL]			10.55(7)		13.56(6)	12.35(6)
log*β* [FeL]	4.61(1)	5.13(1)	3.92(3)	4.38(4)	6.00(7)	4,65(6)
log*β* [FeLH_–1_]	–12.36(2)	–3.49(2)	–4.41(2)	–3.99(7)	–1.86(7)	–3.06(6)
log*β* [FeLH_–2_]		–12.59(1)		–12.48(4)	–10.22(6)	–11.16(4)
log*β* [FeLH_–3_]						
log*β* [FeLH_–4_]				–30.28(4)	–28.83(6)	–29.89(4)
p*K*_*a*_ [FeH_6_L]						
p*K*_*a*_ [FeH_5_L]						
p*K*_*a*_ [FeH_4_L]					4.87	
p*K*_*a*_ [FeH_3_L]					5.86	5.84
p*K*_*a*_ [FeH_2_L]						
p*K*_*a*_ [FeHL]			6.46			
p*K*_*a*_ [FeL]			6.63		7.57	7.70
p*K*_*a*_ [FeLH_–1_]	7.95	8.62	8.33	8.37	7.86	7.71
p*K*_*a*_ [FeLH_–2_]	8.68	9.10		8.49	8.36	8.10
p*K*_*a*_ [FeLH_–3_]						
p*K*_*a*_ [FeLH_–4_]						

a *T* = 298 K, *I* = 0.1 M NaClO_4_, standard deviations on the last digit given in parentheses. Overall stability constants (β) expressed by the equation: β([FeH_*n*_L]^(*n*+2)+^) = [[FeH_*n*_L]^(*n*+2)+^]/[Fe(II)][[L]^n+^][H^+^]^n^. Acid dissociation constants (p*K*_a_) expressed as p*K*_a_ = logβ ([FeH_*n*_L]^(*n*+2)+^) – log β ([FeH_*n*–1_L]^(*n*+1)+^).

For the first four ligands (L1–L4), in which the only deprotonating residue is His, the complexation begins at a pH between 4 and 5 (Table 4 and Figure 2). The first observed complex form differs in the number of deprotonated residues, depending on the number of His residues present in the ligand (two deprotonated imidazole nitrogens in L1 and L4 and three in both L2 and L3). Together with the deprotonation of subsequent His residues, the following species are formed, eventually leading to the formation of fully deprotonated [FeL] species. Most of the deprotonation events are not stepwise; thus, we cannot be certain of the number of imidazole nitrogens bound to the Fe(II) ions. The deprotonation pattern is consistent with previously described polymorphic binding site phenomenon, suggesting the occurrence of this event.

Further deprotonations lead to the formation of species in which up to four additional protons dissociate, most probably from water molecules or amide groups of the peptide backbone. In the literature, there is evidence regarding Fe(II) ion coordination to amide nitrogen of not only peptide ligands but also other systems, such as macrocycles.^45−50^ However, there are scarce sources regarding the p*K*_*a*_ values of amide nitrogen deprotonation for Fe(II)-containing systems. The deprotonation constants presented in this paper are in good agreement with data presented in other recent works on Fe(II) ion complexes with peptide ligands, thus supporting the theory of the complexation of amide nitrogens (Table 4).^45,46^ As we are not able to prove this statement, we can maintain that these additional species may be associated, akin to the rest of the studied Fe(II) complexes, with deprotonation and participation in the binding of amide nitrogen atoms or deprotonation of water molecules.

For both acid-containing L5 and L6 ligands, the complexation occurs at a slightly lower pH of approximately 3–4 (Table 4 and Figure 2). The first complex form of L5, [FeH_5_L]^3+^, arises from the deprotonation of all four Asp residues. In the case of L6, the first species is [FeH_4_L]^2+^, originating from the deprotonation of all Glu residues and one His residue. Once again, this is consistent with the lower acidity of Glu compared to Asp. Further deprotonation involves imidazole nitrogen atoms. In the case of L5 and L6, we can observe the water/amide nitrogen deprotonation as well, resulting in the formation of the [FeLH_–1_]^3–^, [FeLH_–2_]^4–^, and [FeLH_–4_]^6–^ species. The deprotonation pattern is consistent with the previously mentioned polymorphic binding site phenomenon, suggesting the coexistence of various complex forms, differing in the set of binding His residues.

#### Zn(II) Complexes

**Figure 3:**
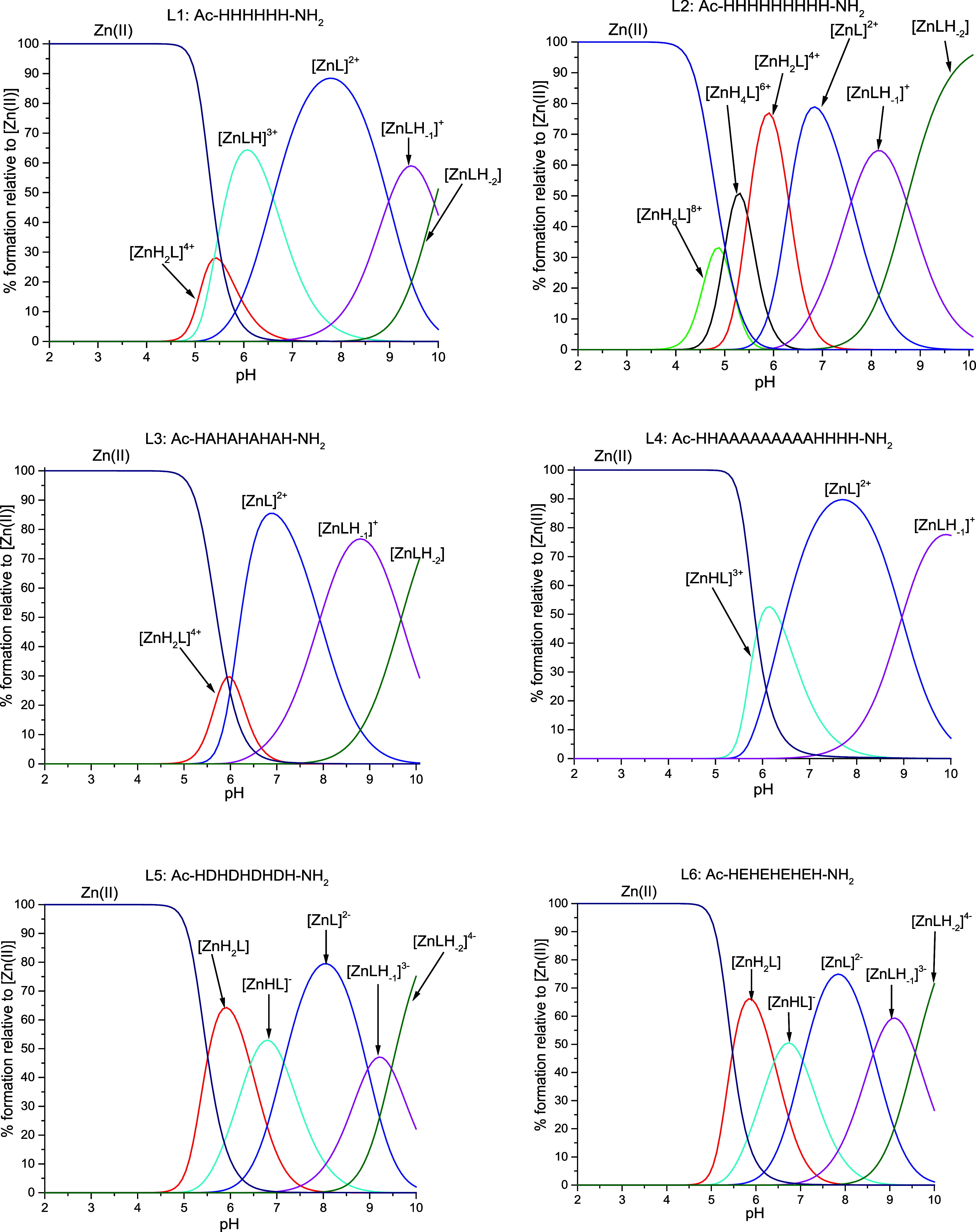
Species distribution diagrams of complexes formed between the Zn(II) and L1–L6 systems. Species distribution calculated for potentiometric titration experimental conditions. [Zn(II)]_tot_= 0.5 mM; M/L = 1:1.1.

For all ligands, calculations based on potentiometric data suggest the formation of mononuclear species with various protonation states (Table 5 and Figure 3).

**Table 5:** Stability Constants (log*β*), and p*K*_*a*_ values of All Zn(II)/Peptide Systems^a^

	ligands					
assignments	L1	L2	L3	L4	L5	L6
log*β* [ZnH_6_L]		43.51(4)				
log*β* [ZnH_5_L]						
log*β* [ZnH_4_L]		33.54(2)				
log*β* [ZnH_3_L]						
log*β* [ZnH_2_L]	19.63(6)	22.61(2)	18.32(2)		21.42(1)	21.03(1)
log*β* [ZnHL]	14.26(3)			13.13(2)	14.99(3)	14.62(3)
log*β* [ZnL]	7.66(5)	9.99(3)	6.72(1)	6.71(4)	7.84(4)	7.58(3)
log*β* [ZnLH_–1_]	–1.32(8)	2.40(5)	–0.86(4)	–2.25(7)	–1.12(5)	–1.06(5)
log*β* [ZnLH_–2_]	–11.24(7)	–6.32(6)	–10.08(4)		–10.59(5)	–10.63(5)
p*K*_*a*_ [ZnH_6_L]						
p*K*_*a*_ [ZnH_5_L]						
p*K*_*a*_ [ZnH_4_L]						
p*K*_*a*_ [ZnH_3_L]						
p*K*_*a*_ [ZnH_2_L]						
p*K*_*a*_ [ZnHL]	5.37				6.43	6.41
p*K*_*a*_ [ZnL]	6.60			6.42	7.15	7.04
p*K*_*a*_ [ZnLH_–1_]	8.98	7.59	7.58	8.96	8.96	8.64
p*K*_*a*_ [ZnLH_–2_]	9.92	8.72	9.22		9.47	9.57

a *T* = 298 K, *I* = 0.1 M NaClO_4_, standard deviations on the last digit given in parentheses. Overall stability constants (β) expressed by the equation: β([ZnH_*n*_L]^(*n*+2)+^) = [[ZnH_*n*_L]^(*n*+2)+^]/[Zn(II)][[L]^*n*+^][H^+^]^*n*^. Acid dissociation constants (p*K*_a_) expressed as p*K*_a_ = log β([ZnH_*n*_L]^(*n*+2)+^) – log β([ZnH_*n*–1_L]^(*n*+1)+^).

For the four systems containing only deprotonating His residues (L1–L4), the coordination pH range is typical for Zn(II) ion complexes in poly-His systems (Table 5 and Figure 3).^33−36^ The deprotonation processes vary between all four described systems, depending on the number of His residues in the sequences. In the first species, three (L1 and L4) or four (L2 and L3) His residues are deprotonated, and most probably at least two His coordinate to the Zn(II) ion. All complexes demonstrate a similar deprotonation pattern, consistent with the previously mentioned polymorphic binding site phenomenon. Eventually, all ligands become fully deprotonated and achieve the [ZnL] form. Further deprotonation of those species provides us with [ZnLH_–1_] and [ZnLH_–2_] forms, arising most probably as the consequence of the deprotonation of one or two water molecules from the Zn(II) ion’s coordination sphere. Due to the lack of spectroscopic data available for this *d*^10^ metal ion, it is difficult to prove the number of imidazoles binding to the central metal ions. We can speculate that in each of the species, two to three His residues are involved in Zn(II) coordination. Potentiometric titrations of the Zn(II) complex with a peptide containing nine adjacent His residues (comparable to L2) were already performed by Watly *et al*. The studies resulted in the discovery of four complex forms, each coordinated by three His residues.^32^ Although the peptide studied by Watly *et al*. possesses additional amino acids not involved in Zn(II) binding next to the poly-His region, the obtained stability constants remain in very good agreement with log*β* presented in this work (Table 5). On the basis of the similarities between stability constants, we can suppose {3N_im_} coordination in the entire studied pH range.^32^ Taking into account the high number of possible donor groups as well as the conformational flexibility of Zn(II), the formation of a mixture of complex forms with different geometries could be observed. The geometry of Zn(II) complexes can vary from tetra- to penta-coordinated systems with distorted tetrahedral or pyramidal arrangements, respectively.^51,52^

For the two acid-containing L5 and L6 ligands, the complexation process begins at pH 4.5, with the deprotonation of four acidic residues and three His residues (Table 5 and Figure 3). For both systems, the first observed complex form is [ZnH_2_L]. Further deprotonation patterns are comparable to those of the L1–L4 systems.

### Comparison of the Thermodynamic Stabilities of Metal Complexes

The stability constants (log*β*) directly describe the binding power between the ligands and metal ions. Despite being a good indicator of complex stability, they cannot be compared between ligands containing various binding groups, as the differences in the ligand deprotonation constants may affect the log*β*/p*K*_*a*_ values. To accurately compare the metal chelation affinity of examined peptides with each other and with Mn(II), Fe(II), and Zn(II) ions, we have used a variety of different tools.

**Figure 4:**
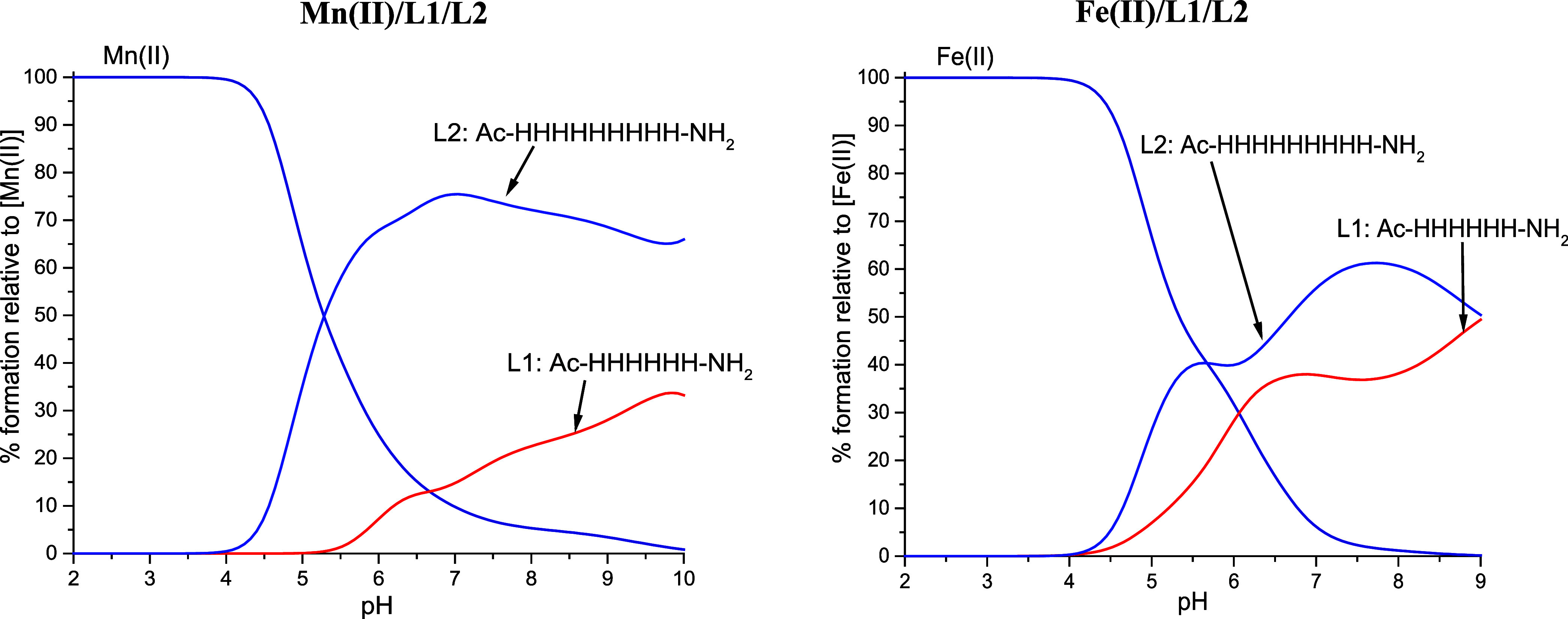
Competition plots between the L1/L2 ligands and metal ions. The plot describes the hypothetical situation in which equimolar amounts of all reagents are mixed. The left plot presents Mn(II) systems, and the right plot presents Fe(II) systems. Conditions: *T* = 298 K, *I* = 0.1 M NaClO_4_; the concentration of all reagents is 1 × 10^–3^ M.

**Figure 5:**
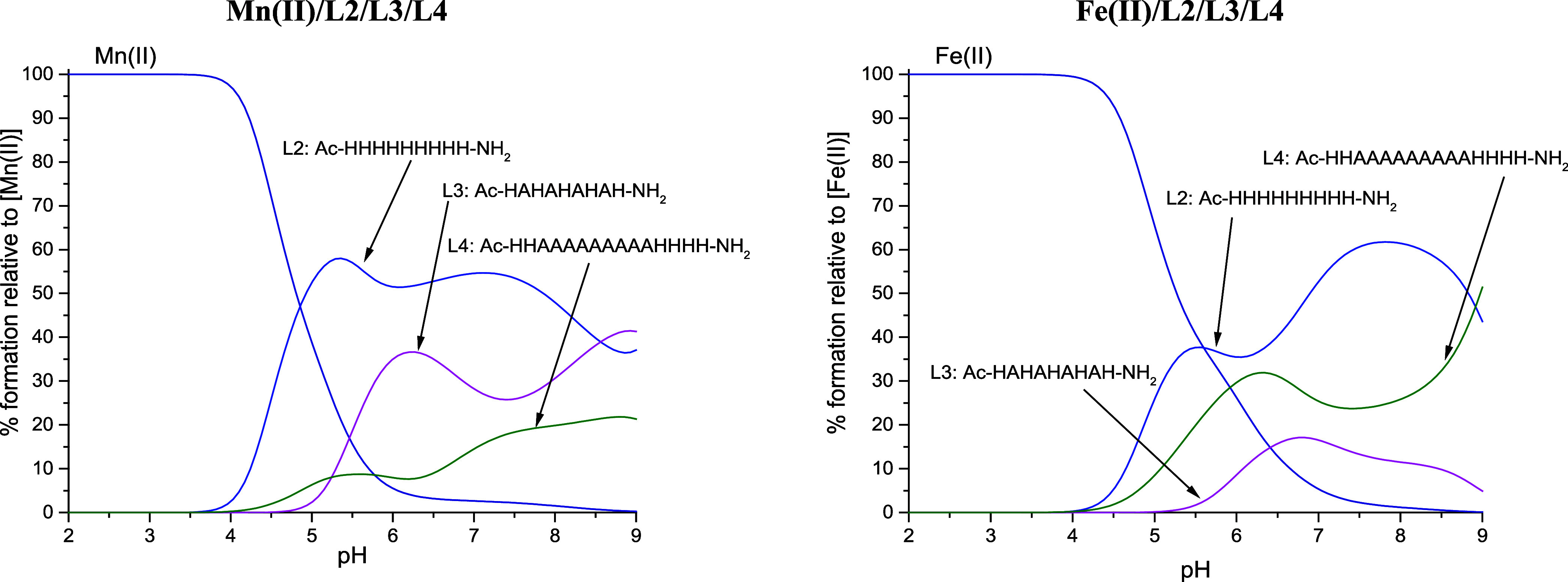
Competition plots between the L2/L3/L4 ligands and metal ion. The plot describes the hypothetical situation, in which equimolar amounts of all reagents are mixed. The left plot presents Mn(II) systems, and the right plot presents Fe(II) systems. Conditions: *T* = 298 K, *I* = 0.1 M NaClO_4_; the concentration of all reagents is 1 × 10^–3^ M.

**Figure 6:**
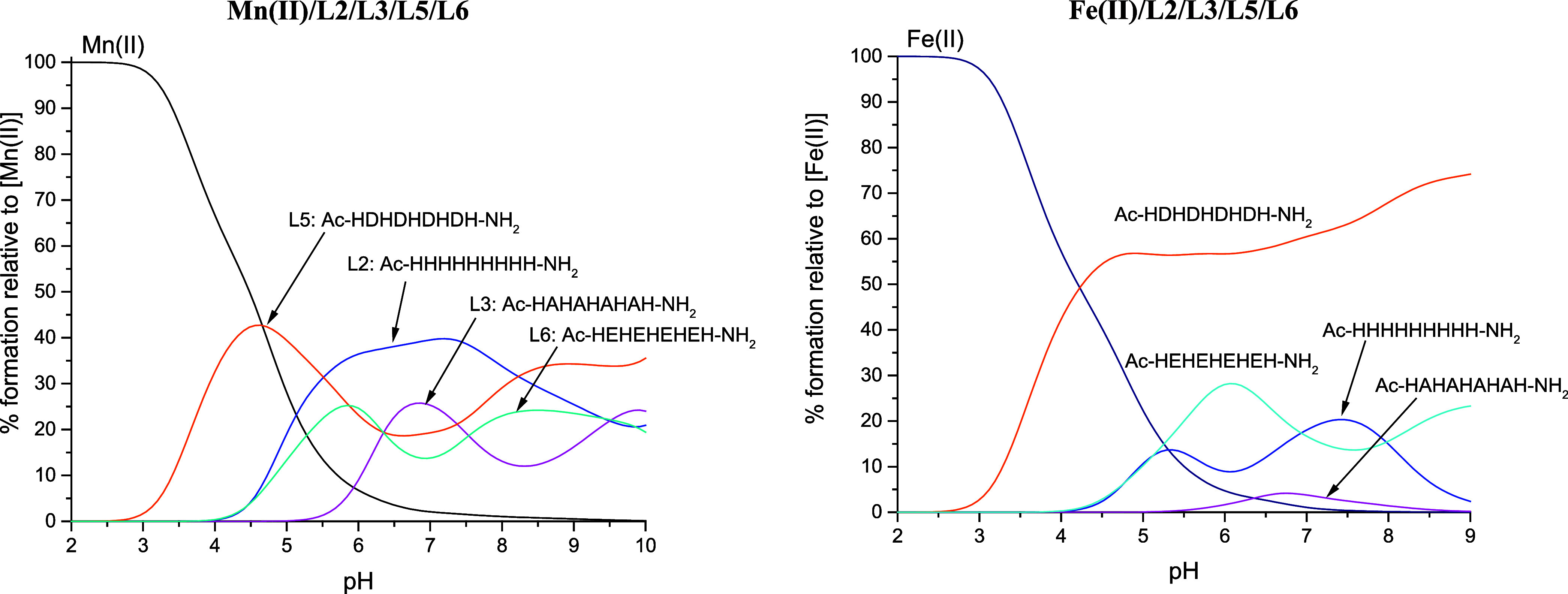
Competition plots between the L2/L3/L5/L6 ligands and the metal ion. The plot describes the hypothetical situation, in which equimolar amounts of all reagents are mixed. The left plot presents Mn(II) systems, and the right plot presents Fe(II) systems. Conditions: *T* = 298 K, *I* = 0.1 M NaClO_4_; the concentration of all reagents is 1 × 10^–3^ M.

The first method of assessing the metal chelation efficacy is a direct comparison of competition plots between the ligands and selected metal ions. This method displays a hypothetical situation in which equal concentrations of all reagents are present in the solution. We have chosen to compare the stability of various systems based on the position and number of His, Asp, and Glu residues in the ligands. The selected competitions are L1/L2—to assess the influence of the number of binding His residues on the stability of complexes (Figure 4); L2/L3/L4—to understand the influence of His position and separation on complex stability (Figure 5); and finally, L2/L3/L5/L6—to examine the influence of the introduction of carboxylic acid residues into poly-His peptide sequences (Figure 6).

#### L1/L2

For all metal ions and in the entire pH range, the competition plot clearly indicates a higher efficiency of Mn(II) complexation by the longer, nine-His L2, over the shorter six-His L1. For all discussed metal ions, L2 binding starts at a lower pH, which is consistent with the acidity of deprotonated His residues (Table 2), and forms the most stable complexes. The rise in stability of L2 metal complexes may be correlated with the number of polymorphic states in which the system can exist—the more residues, the more possibilities for metal ion coordination.^32^ Additionally, the strong preference for the longer L2 may come from the lack of steric strain when assuming a preferred octahedral coordination geometry and the number of His bound to the metal ion. In the case of L1, most probably, the preferred coordination would impose steric strain, making the complex less energetically favorable.

The smallest difference in metal chelation affinity can be observed in the case of the Fe(II) ions. The preference for L1 and L2 is close to equal, especially under higher pH conditions (above 8). This could possibly be explained by the ability of Fe(II) ions to bind to amide nitrogens of the peptide backbone, as the only metal ion in this study. The pH at which the binding strengths of both ligands start to be comparable corresponds to the pH of amide deprotonation and participation in the Fe(II) binding.

For Zn(II), all of the presented competition plots reveal L2 as the strongest ligand. This behavior is typical for Zn(II) ions and is in agreement with broad literature data; thus, we do not discuss it in detail and only show competition plots in Figure S11.^33−36^ Overall, the combination of strong preferences of Zn(II) to form complexes with borderline donor atoms such as nitrogen, the length of the peptide, availability of multiple binding sites, and position of His residues work in favor of the stability of the Zn(II)/L2 complex.

#### L2/L3/L4

The competition between L2/L3/L4 ligands bearing solely His as coordinating residues shows that L2, consisting of nine adjacent His residues, forms the most stable complexes across almost the entire pH range and for all studied metal ions. The separation of His residues in the peptide sequence affects the ligand’s affinity toward Mn(II) and Fe(II) in different ways. L3, with five His residues each separated by one Ala residue, forms stronger complexes with Mn(II) than L4, which consists of six His residues separated by a long poly-Ala chain. Most likely, the separation by Ala makes the His residues more available for the metal ion, promoting polymorphic binding and increasing the number of possible coordination modes. On the other hand, the presence of a long flexible chain separating possible binding sites decreases the stability of Mn(II) ion complexes. The most probable explanation is the fact that Mn(II) ions do not displace protons from the amide group and can bind to the peptide only via imidazole nitrogen atoms. Thus, the increase in the distance between two anchoring sites reduces the overall stability of macrochelated complexes due to the possible presence of a large poly-Ala loop in the formed species.

For Fe(II) ions, the tendency between L3 and L4 is reversed, with a more stable complex being formed with the L4 ligand (Figure 5). The possibility of amide nitrogen deprotonation may be responsible for such behavior, decreasing the influence of the long poly-Ala chains on the stability of complexes. As the Ala side chain is small and uncharged, after binding to anchoring imidazoles to complete their coordination sphere, Fe(II) ions have easy access to amide nitrogens of neighboring residues.

#### L2/L3/L5/L6

In this competition, we aim to reflect the influence of the introduction of carboxylic acids into the sequences of poly-His peptides. For Mn(II) ions, below a pH of 5.5, there is a distinct preference for Asp residues containing L5, which could be easily explained when we compare the deprotonation constants of the ligands (Table 2). Together with the deprotonation of His residues of L2, an increase in the stability of this system is observed, and above pH 5.5, the most stable complexes are formed with the L2 ligand. Acid-containing peptides form weaker complexes than peptides containing only His. At pH 5.5, five His residues are deprotonated in the case of L2, while only one His residue loses the proton in the case of L5 and L6. At pH 7, L2 possesses eight deprotonated His residues that are able to bind Mn(II) ions, while L5 and L6 have just three deprotonated His residues. Thus, the rise in stability of L2 complexes once again may be correlated with the number of possible binding sites and polymorphic states in which the system can exist—the higher the number of deprotonated His residues, the more possibilities for metal ion coordination. The same explanation can be used in the case of L3, which forms more stable complexes than L5 and L6 under a narrow pH range of 6.3–7.4.

For Fe(II) ions, we observe different behavior. The most stable complexes are formed with the Asp-containing L5 ligand throughout the entire pH range. Although the Fe(II) ion is considered a borderline acid, there is an abundance of hard oxygen donors in Fe(II) binding sites of proteins, which confirms the possibility of such binding.^6^ Surprisingly, the type of acid present in the sequence is of great importance to the stability of the complex. Complexes of L6, containing Glu, are less competitive with other ligands compared to Asp-containing L5. This might be based on the earlier deprotonation of Asp residues in relation to Glu residues (Table 2), together with the steric effects resulting from the differences in amino acid side chain length. The comparison becomes far less trivial in the cases of L2 and L6. L6 complexes are more stable than complexes with L2 up to pH 7, when the stability of L2 rises and surpasses L6. The higher stability of L6 up until pH 7 can be explained by the negative charge of the deprotonated Glu residues, partially responsible for added stabilization by decreasing the overall charge of the complex.^18^ Around pH 7, all 9 His residues of L2 become deprotonated, while in L6 only 3 His residues are deprotonated. The higher number of deprotonated His residues allows for more possibilities of polymorphic binding of L2 and thus aids the increased complex stability. In peptides, the pH at which amides deprotonate depends on the number of His present in the studied ligand. The general rule is the more His, the higher the p*K*_a_ of amide deprotonation.^35,53^ Around pH 7.6, the amide deprotonation begins in L6 (5 His), starting the gradual increase of the complex stability, and around pH 8 surpassing L2 (9 His), in which the amide deprotonation starts around pH 8.6 (Table 2). The L3 peptide forms the weakest complexes among all peptides considered in this competition.

Although the competition plots are a great tool for understanding the coordination preferences of Mn(II), Fe(II), and Zn(II) ions, to compare the stability of the ligands under *in vivo* conditions, it is worth calculating the dissociation constant (*K*_d_) of each studied system. The dissociation constant is the concentration of free metal ion (mol/dm^3^) in the situation where half of the studied ligand is bound to a metal ion, and the other half is not complexed.^54^*K*_d_ does not depend on the concentration of reagents and refers to the general complex formation equilibrium M + L = ML. The constant is pH-dependent. The lower the *K*_d_ value, the stronger the complex.

The studied systems are based on motifs found in various naturally occurring Mn II), Fe(II), and Zn(II) transporters in bacteria, fungi, and plants. Dissociation constants for some of the most prevalent systems have been collected in Table 6. Although some of those *K*_d_ values have been calculated under slightly different pH conditions, the general pH range of all calculations is 7–7.5 and lies within the physiological pH of the cell, thus enabling us to compare the binding strength of those ligands. It has to be underlined that the comparison between short model peptides and full proteins is not the most fortunate one and can be just indicative. The difference in the length, tertiary structure, and the presence of additional weak interactions, such as hydrogen bonds in proteins, can stabilize the complex much further than in model peptides. These effects may greatly increase the stability of formed complexes and their dissociation constants. But since our studies are systemic and aim to shed light on the insufficiently discovered coordination chemistry of Mn(II) and Fe(II) ions, we have decided to include those comparisons.

**Table 6:** Comparison of *K*_d_ Values for Studied and Biological Ligands for Mn(II), Fe(II), and Zn(II) Ions^a^

ligand	Mn(II)	Fe(II)	Zn(II)	reference
L1: Ac-HHHHHH-NH_2_	5.35 × 10^–4^	9.46 × 10^–5^	7.29 × 10^–8^	this work
L2: Ac-HHHHHHHHH-NH_2_	3.07 × 10^–5^	4.28 × 10^–5^	5.64 × 10^–10^	this work
L3: Ac-HAHAHAHAH-NH_2_	6.05 × 10^–5^	2.56 × 10^–4^	1.83 × 10^–6^	this work
L4: Ac-HHAAAAAAAAAHHHH-NH_2_	3.80 × 10^–4^	1.49 × 10^–4^	8.32 × 10^–7^	this work
L5: Ac-HDHDHDHDH-NH_2_	8.41 × 10^–5^	6.27 × 10^–6^	1.77 × 10^–7^	this work
L6: Ac-HEHEHEHEH-NH_2_	1.25 × 10^–4^	1.88 × 10^–5^	1.49 × 10^–7^	this work
*Escherichia coli* Feo B fragments	7.02 × 10^–7^	4.75 × 10^–7^	6.31 × 10^–8^	(45)
*Escherichia coliFur*	2.4 × 10^–5^	1.2 × 10^–6^	1.4 × 10^–10^	(55)
*Streptococcus pyogenes* MtsA		4.3 × 10^–6^		(56)
*Bacillus subtilis* MntR	2 × 10^–7^–2 × 0^–6^			(57)
*Yersinia pestis* YfeA	1.78 × 10^–8^		6.6 × 10^–9^	(58)
*Treponema pallidum* TroA	7.1 × 0^–9^		2.25 × 10^–8^	(59)
*Deinococcus radiodurans* MntH	1.9 × 10^–4^			(60)
*Synechocystis* ZnuA			7.3 × 10^–9^	(61)

a *K*_d_ values calculated as:  at pH = 7.0.

The dissociation constants of all studied peptide systems are in the range of the weakest bacterial proteins, transporting Mn(II), Fe(II), and Zn(II), as reported in Table 6. The results obtained from *K*_d_ calculations remain in good agreement with the previously presented competition plots. Of all studied Mn(II) ligands, L2 appears to be the strongest at neutral pH (7.0). In the case of Fe(II) ions, L5 has proven to form the most stable complexes in comparison to other studied systems. The strongest chelator of Zn(II) ions proves to be L2. In addition, the comparison of *K*_d_ values shows that the affinity of Zn(II) ions for all studied ligands is distinctly higher than for Mn(II) and Fe(II) ions, which is in accordance with the Irving-Williams series. In the case of L2 and L3, the Mn(II) affinity at pH 7.0 is slightly higher than the affinity for Fe(II), but for L1, L4, L5, and L6, the opposite trend is observed.

## Conclusions

Knowledge of the coordination chemistry of biologically important transition metals is necessary to better understand the function of proteins involved in the homeostasis of metal ions. The results presented in this work have an important impact on the field of Mn(II) and Fe(II) coordination chemistry and are among the first solution studies of Mn(II) and Fe(II)/peptide complexes.

Detailed analysis of the results obtained by the use of spectrometric, spectroscopic, and potentiometric techniques has shown that all of the studied ligands possess efficient Mn(II), Fe(II), and Zn(II) binding motifs. For all investigated systems, the coexistence of various mononuclear polymorphic species with different sets of imidazole nitrogen donors was found. When the thermodynamic stability of the studied ligands is compared, numerous implications on the influence of the occurrence, position, and number of binding residues might be drawn. (a) The elongation of the poly-His chain increased the efficiency of Mn(II), Fe(II), and Zn(II) ion binding. (b) The separation of His residues by nonbinding residues (Ala) decreased the stability of Mn(II), Fe(II), and Zn(II) complexes. In the case of Mn(II) and Zn(II) ions, which do not displace hydrogen from the amide bond, the peptide containing neighboring His and Ala residues (L3) exhibited higher binding affinity than the peptide containing a long poly-Ala chain separating the binding His residues (L4). For Fe(II) ions, the only one among the studied ions capable of deprotonating amides, the opposite trend is observed. (c) The presence of oxygen donor atoms in poly-His peptide sequences does not have a major impact on the ligand affinity toward Mn(II) ions. The presence of multiple possibilities of binding, presented as “so-called” polymorphic binding sites, proved to increase the stability of formed complexes more than the mere presence of preferred oxygen donors. In the case of Fe(II) ions, the type of acidic residue is of great importance to the stability of the formed complexes. The most stable complexes are formed with the ligand containing Asp residues (L5).

Overall, there is an urgent need for broader systematic studies, which would help in better understanding the preferences and patterns governing the coordination chemistry of Mn(II) and Fe(II) ions in proteins. Studies of further peptides, being direct fragments of Mn(II) and Fe(II) proteins, are underway in our laboratory.

## Supporting Information Available

Picture of the titration vessel before and after exposure to air, hydrolysis constants used for potentiometric calculations, distributions diagrams of all L1–L6 ligands deprotonation, ESI-MS spectrum of M(II)/L5 systems taken in different metal/ligand ratios, an exemplary ESI-MS spectrum of Mn(II)/L2 system, a table of intensity maxima of all major complexes and adduct ions observed by ESI-MS, RT EPR spectra of all studied systems, competition plots for Zn(II) ions (PDF)
