# Supplementary material for: Systematic Model Peptide Studies: A Crucial Step To Understand the Coordination Chemistry of Mn(II) and Fe(II) in Proteins
Source: Inorg Chem. 2025 Mar 11;64(11):5472–86. doi: 10.1021/acs.inorgchem.4c05380 (PMC11938343; doi:10.1021/acs.inorgchem.4c05380)
Supplement: Supplementary file 1 — ic4c05380_si_001.pdf [file ic4c05380_si_001.pdf]

Supporting information for:

Systematic model peptide studies - a crucial step to understand the coordination chemistry of Mn(II) and Fe(II) in proteins

Karolina Pawlik, Malgorzata Ostrowska* and Elzbieta Gumienna-Kontecka

Complete affiliations and orcids:

Corresponding Author:

Malgorzata Ostrowska: Faculty of Chemistry, University of Wrocław, 50-383 Wrocław, Poland; https://orcid.org/0000-0001-9765-8914; e-mail: malgorzata.ostrowska3@uwr.edu.pl

Authors:

Karolina Pawlik: Faculty of Chemistry, University of Wrocław, 50-383 Wrocław, Poland; https://orcid.org/0000-0002-3002-4105

Elzbieta Gumienna-Kontecka: Faculty of Chemistry, University of Wrocław, 50-383 Wrocław, Poland; https://orcid.org/0000-0002-9556-6378

a) b)

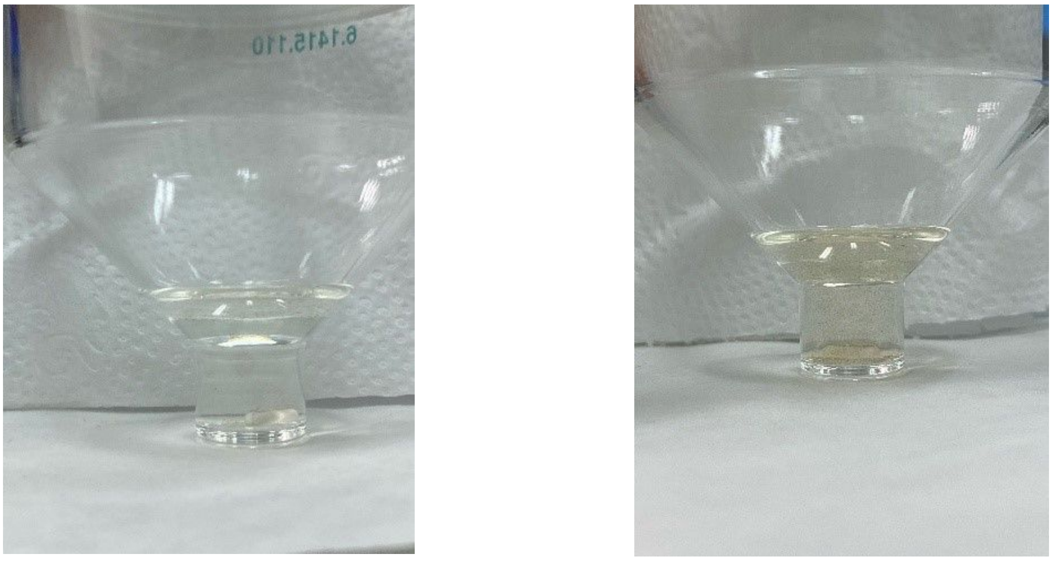

Figure S1. Two pictures presenting the oxidation of white sediment $Mn(OH)_2$ to MnO_2 after potentiometric measurement (pH=11); a) The potentiometric cell immediately after opening b) The potentiometric cell a few minutes after opening.

Table S1. Hydrolysis constants for Mn(II), Zn(II) and Fe(II) ions for I=0.1M ionic strength, T= 298 K. The hydrolysis constants for zero ionic strength were taken from the "Hydrolysis of Metal Cations" by Brown and Ekberg and calculated to 0.1 M ionic strength with the formula proposed by Baes and Mesmer in "The Hydrolysis of Cations".

species	Log β
**Mn(II)**	
$Mn(OH)^+$	-10.78
$Mn(OH)_2$	-22.39
$Mn(OH)_3^-$	-34.34
$Mn(OH)_4^{2-}$	-47.82
**Fe(II)**	
$Fe(OH)^+$	-9.63
$Fe(OH)_2$	-20.73
$Fe(OH)_3^-$	-32.68
**Zn(II)**	
$Zn(OH)^+$	-9.12
$Zn(OH)_2$	-18.08
$Zn(OH)_3^-$	-27.97
$Zn(OH)_4^{2-}$	-39.50

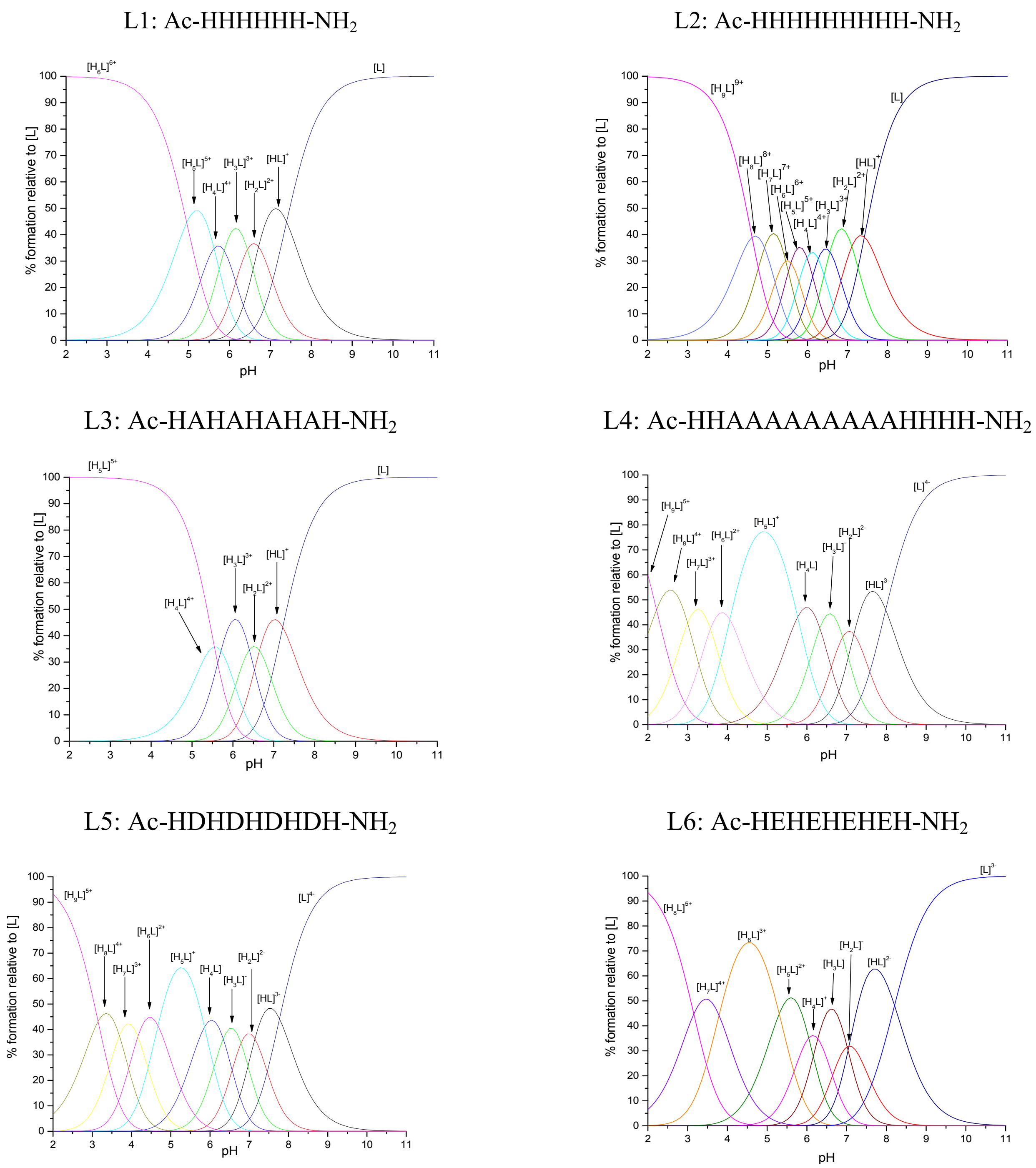

Figure S2. Distribution diagram for L1-L6 deprotonation, calculated for potentiometric titration experimental conditions. $[L]_{tot}$=0.5 mM; 0.1M $NaClO_4$

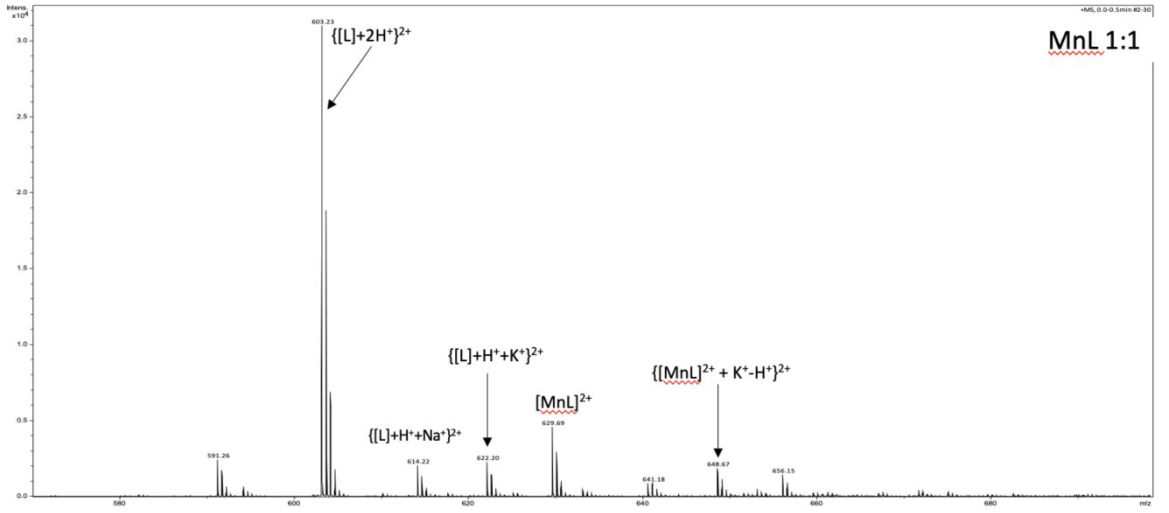

MnL 1:1
$\{[L]+2H^+\}^{2+}$
$\{[L]+H^++Na^+\}^{2+}$
$\{[L]+H^++K^+\}^{2+}$
$[MnL]^{2+}$
$\{[MnL]^{2+} + K^+-H^+\}^{2+}$
603.23
591.26
614.22
622.20
629.69
641.18
648.67
656.15

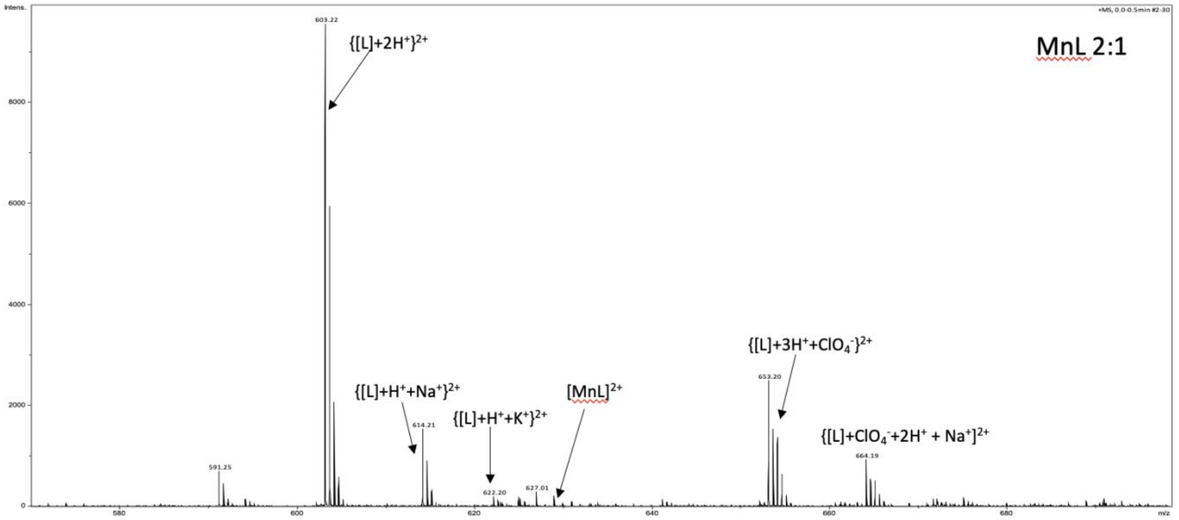

MnL 2:1
$\{[L]+2H^+\}^{2+}$
$\{[L]+H^++Na^+\}^{2+}$
$\{[L]+H^++K^+\}^{2+}$
$[MnL]^{2+}$
$\{[L]+3H^++ClO_4^-\}^{2+}$
$\{[L]+ClO_4^-+2H^+ + Na^+]^{2+}$
603.22
591.25
614.21
622.20
627.01
653.20
664.19

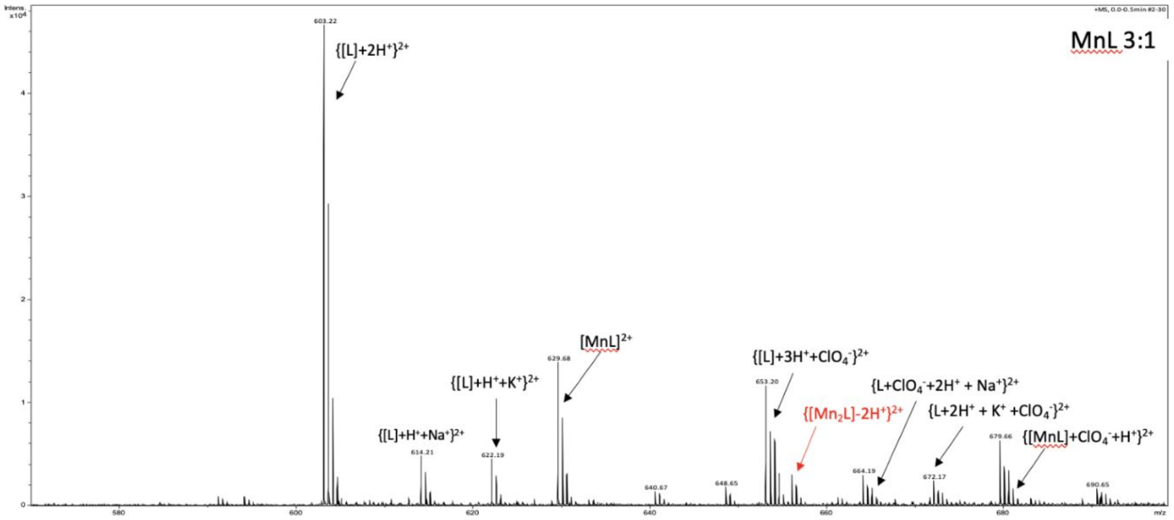

MnL 3:1
$\{[L]+2H^+\}^{2+}$
$\{[L]+H^++Na^+\}^{2+}$
$\{[L]+H^++K^+\}^{2+}$
$[MnL]^{2+}$
$\{[L]+3H^++ClO_4^-\}^{2+}$
$\{[Mn_2L]-2H^+\}^{2+}$
$\{L+ClO_4^-+2H^+ + Na^+\}^{2+}$
$\{L+2H^+ + K^+ +ClO_4^-\}^{2+}$
$\{[MnL]+ClO_4^-+H^+\}^{2+}$
603.22
614.21
622.19
629.68
640.67
648.65
653.20
664.19
672.17
679.66
690.65

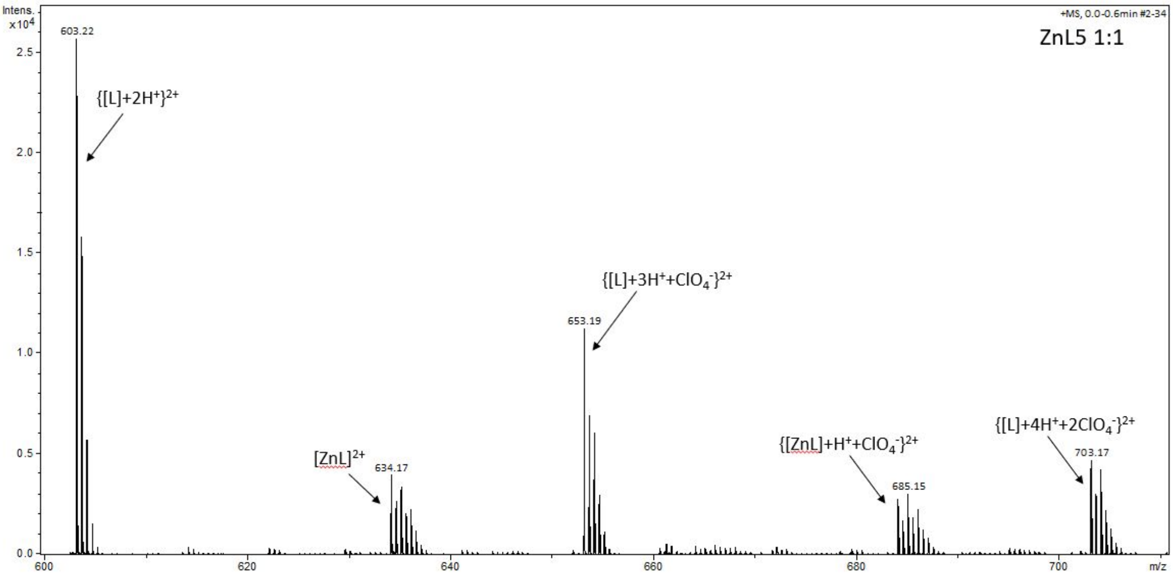
Intens.
x10^4
+MS, 0.0-0.6min #2-34
ZnL5 1:1
603.22
{[L]+2H+}2+
{[L]+3H++ClO4-}2+
653.19
[ZnL]2+
634.17
{[ZnL]+H++ClO4-}2+
685.15
{[L]+4H++2ClO4-}2+
703.17
m/z

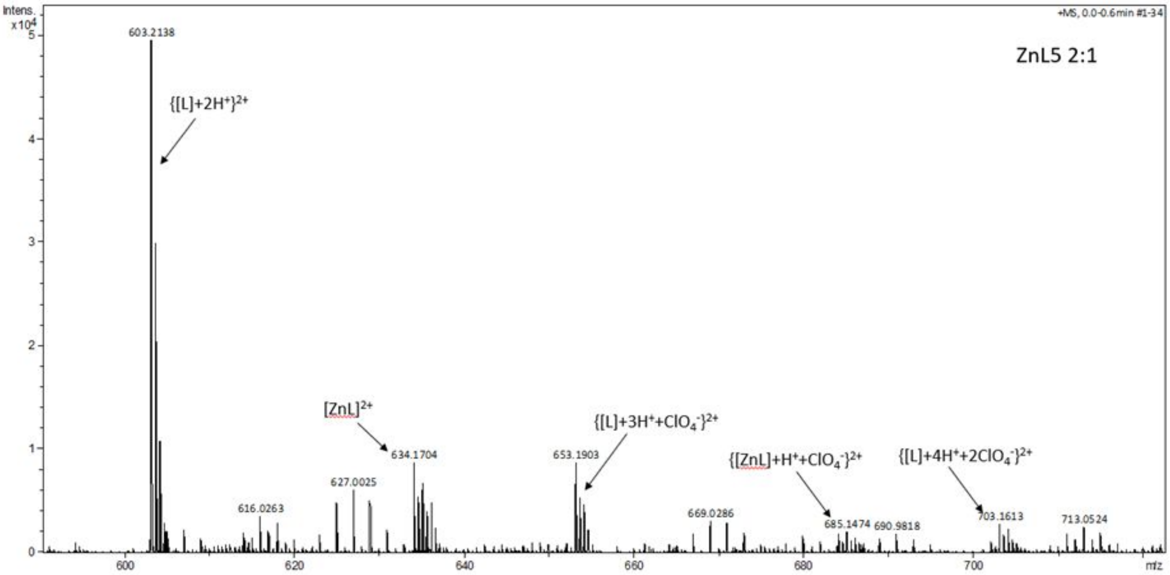
Intens.
x10^4
+MS, 0.0-0.6min #1-34
ZnL5 2:1
603.2138
{[L]+2H+}2+
[ZnL]2+
634.1704
{[L]+3H++ClO4-}2+
653.1903
{[ZnL]+H++ClO4-}2+
{[L]+4H++2ClO4-}2+
616.0263
627.0025
669.0286
685.1474
690.9818
703.1613
713.0524
m/z

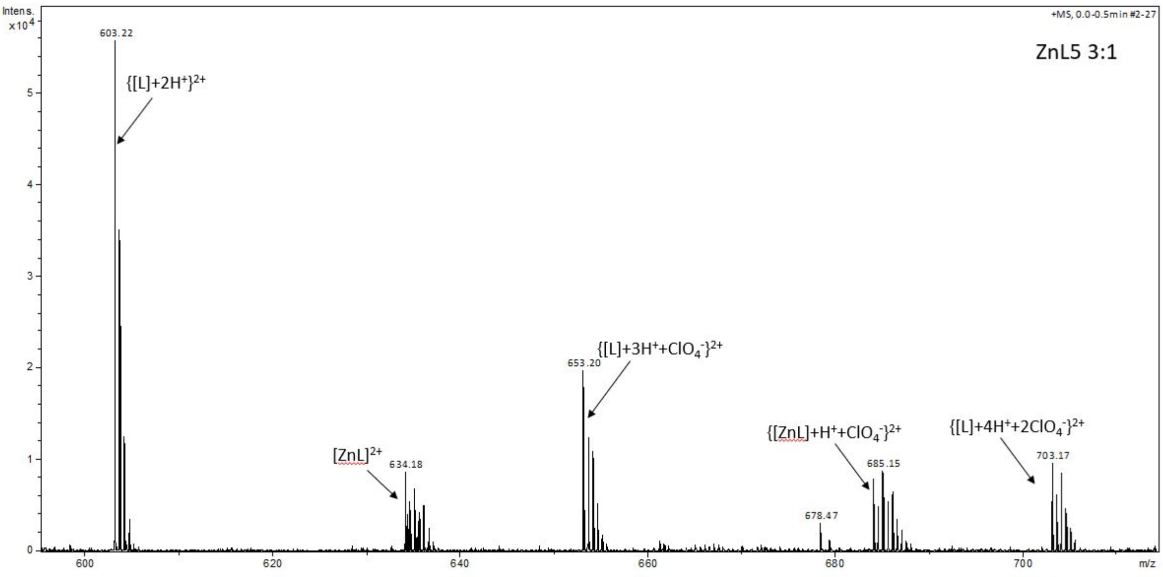
Intens.
x10^4
+MS, 0.0-0.5min #2-27
ZnL5 3:1
603.22
{[L]+2H+}2+
653.20
{[L]+3H++ClO4-}2+
{[ZnL]+H++ClO4-}2+
{[L]+4H++2ClO4-}2+
[ZnL]2+
634.18
685.15
703.17
678.47
m/z

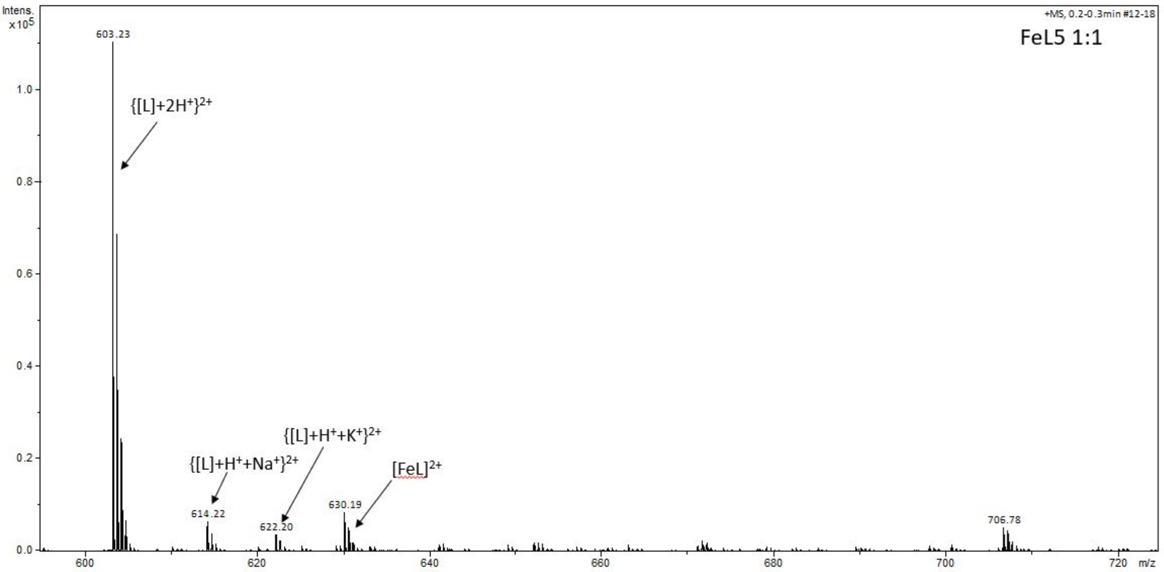

Intens.
x10^5
+MS, 0.2-0.3min #12-18
FeL5 1:1
603.23
{[L]+2H+}2+
{[L]+H+ +Na+}2+
{[L]+H+ +K+}2+
[FeL]2+
614.22
622.20
630.19
706.78
m/z

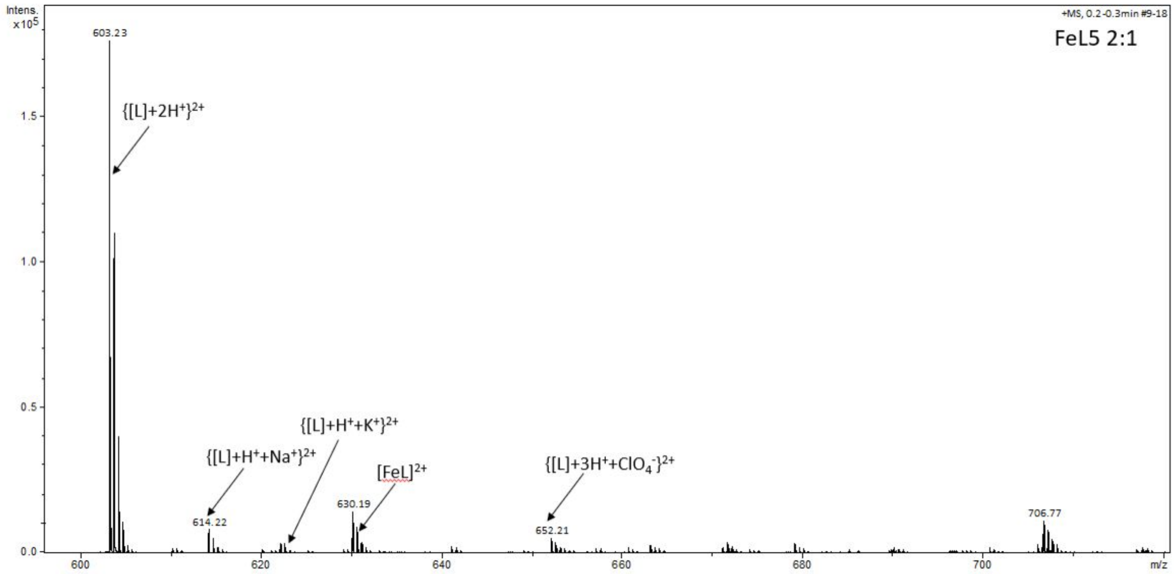

Intens.
x10^5
+MS, 0.2-0.3min #9-18
FeL5 2:1
603.23
{[L]+2H+}2+
{[L]+H+ +Na+}2+
{[L]+H+ +K+}2+
[FeL]2+
{[L]+3H+ +ClO4-}2+
614.22
630.19
652.21
706.77
m/z

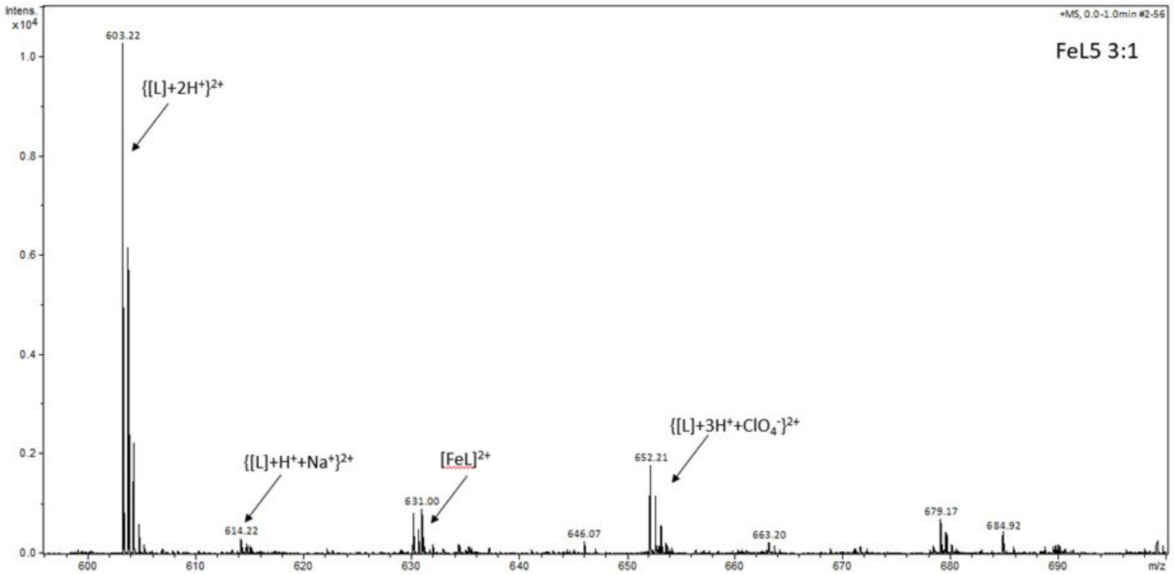

Intens.
x10^4
+MS, 0.0-1.0min #2-56
FeL5 3:1
603.22
{[L]+2H+}2+
{[L]+H+ +Na+}2+
[FeL]2+
{[L]+3H+ +ClO4-}2+
614.22
631.00
646.07
652.21
663.20
679.17
684.92
m/z

Figure S3. ESI-MS spectra for M(II)/L5 system at 1:1, 2:1 and 3:1 (metal:ligand) ratios. The polynuclear species present in the 3:1 (metal:ligand) MnL5 spectrum is marked in red.

The high number of possible metal binding sites may allow the formation of di- or poly-nuclear complexes. As we mentioned in the publication, we have not observed polynuclear species for any of studied ligands in 1:1.1 and 1:2 (metal:ligand) ESI-MS experiments. Taking the valid concerns of the presence of the polynuclear species into consideration we have conducted additional ESI-MS experiments, in which we have obtained spectra of our systems in 1:1, 2:1, and 3:1 (metal:ligand) ratio (for all studied metal ions), and in three different pH (3, 6, 9), adjusted by the addition of 0.1M HCl, or 0.1M NH_4OH. We have analyzed recorded spectra very carefully and found very weak peaks associated with the formation of $[M_2L]^{2+}$ species exclusively in 3:1 (metal:ligand) spectra of MnL complexes (Fig. S2.). We are aware that the intensity of the MS signals does not correlate with its concentration; it correlates with the ability of the molecule to ionize. Here we compare the signals of the same molecule of the same charge – $[MnL]^{2+}$ and $[Mn_2L]^{2+}$ – so their ability of ionization should be quite the same, and the intensity comparison is sensible. It can give the partial information about the abundance of these particular forms.

Taking into account, that we did not observe signals of polynuclear species on 1:1 ESI-MS, our spectra and potentiometric titrations were performed only for 1:1 ratio. We decided to include mononuclear species in potentiometric calculations. We are not able to perform potentiometric titration in an excess of metal ions due to the hydrolysis and precipitation of insoluble precipitate. When we were trying to include the presence of polynuclear species in the models in the Hyperquad calculations we did not achieve acceptable fitting of experimental and calculated curves - these forms must remain ignored, because the program had rejected them.

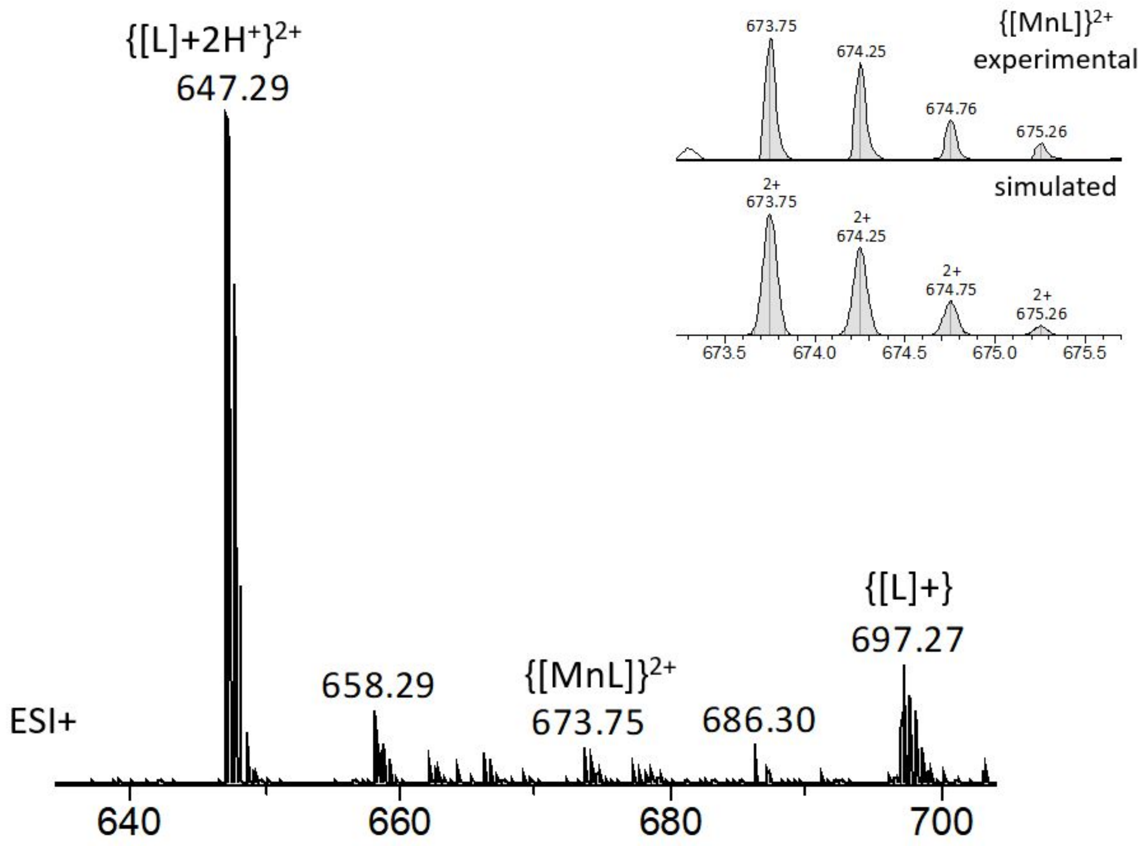

Figure S4. ESI-MS spectrum of Mn(II)/L2 system at 1/1.1 (metal/ligand) ratio. The experimental and simulated isotopic distribution spectra of the peak at *m*/*z* are shown in the upper right corner.

Table S2. Intensity maxima of the major complexes and adduct ions observed by ESI-MS for each M(II)/peptide system, M:L=1:1.1.

**System**	**Ion**	**m/*z* experimental**	**m/*z* simulated**
Mn(II):L1	$\{[L]+H^+\}^+$	882.40	882.40
	$\{[L]+2H^+\}^{2+}$	441.66	441.70
	$\{[L]+3H^+\}^{3+}$	294.72	294.80
	$\{[MnL]^{2+}\}$	468.13	468.16
	$\{[MnL]^{2+}+H^+\}^{3+}$	312.37	312.44
Mn(II):L2	$\{[L]+2H^+\}^{2+}$	647.29	647.29
	$\{[L]+3H^+\}^{3+}$	431.86	431.86
	$\{[L]+4H^+\}^{4+}$	324.15	324.15
	$\{[MnL]^{2+}\}$	673.75	673.75
	$\{[MnL]^{2+}+H^+\}^{3+}$	449.50	449.50
Mn(II):L3	$\{[L]+H^+\}^+$	1029.49	1029.49
	$\{[L]+2H^+\}^{2+}$	515.22	515.25
	$\{[MnL]^{2+}\}$	541.68	541.71
	$\{[MnL]^{2+}+H^+\}^{3+}$	361.41	361.47

Mn(II):L4	$\{[L]+H^{+}\}^{+}$	1521.73	1521.73
	$\{[L]+2H^{+}\}^{2+}$	507.92	507.92
	$\{[L]+3H^{+}\}^{3+}$	761.37	761.37
	$\{[L]+4H^{+}\}^{4+}$	381.19	381.19
	$\{[MnL]^{2+}\}$	787.83	787.83
	$\{[MnL]^{2+}+H^{+}\}^{3+}$	525.56	525.56
Mn(II):L5	$\{[L]+H^{+}\}^{+}$	1205.45	1205.45
	$\{[L]+2H^{+}\}^{2+}$	603.21	603.23
	$\{[L]+3H^{+}\}^{3+}$	402.44	402.49
	$\{[MnL]^{2+}\}$	629.67	629.69
	$\{[MnL]^{2+}+H^{+}\}^{3+}$	420.08	420.13
Mn(II):L6	$\{[L]+H^{+}\}^{+}$	1261.52	1261.51
	$\{[L]+2H^{+}\}^{2+}$	631.24	631.26
	$\{[L]+3H^{+}\}^{3+}$	421.13	421.17
	$\{[MnL]^{2+}\}$	657.71	657.72
	$\{[MnL]^{2+}+H^{+}\}^{3+}$	438.77	438.82
Fe(II):L1	$\{[L]+H^{+}\}^{+}$	882.39	882.40
	$\{[L]+2H+\}^{2+}$	441.70	441.70
	$\{[L]+3H^{+}\}^{3+}$	294.80	294.80
	$\{[FeL]^{2+}\}$	467.66	467.66
Fe(II):L2	$\{[L]+2H^{+}\}^{2+}$	647.29	647.29
	$\{[L]+3H^{+}\}^{3+}$	431.86	431.86
	$\{[L]+4H^{+}\}^{4+}$	324.15	324.15
	$\{[FeL]^{2+}\}$	674.25	674.25
	$\{[FeL]^{2+}+H^{+}\}^{3+}$	449.84	449.84
	$\{[FeL]^{2+}+2H^{+}\}^{4+}$	337.63	337.63
Fe(II):L3	$\{[L]+H^{+}\}^{+}$	1029.48	1029.49
	$\{[L]+2H^{+}\}^{2+}$	515.25	515.25
	$\{[L]+3H^{+}\}^{3+}$	343.83	343.83
	$\{[FeL]^{2+}\}$	542.21	542.21
	$\{[FeL]^{2+}+H^{+}\}^{3+}$	361.81	361.82
Fe(II):L4	$\{[L]+H^{+}\}^{+}$	1521.73	1521.73
	$\{[L]+2H^{+}\}^{2+}$	761.36	761.37
	$\{[L]+3H^{+}\}^{3+}$	507.91	507.92
	$\{[L]+4H^{+}\}^{4+}$	381.13	381.19
	$\{[FeL]^{2+}\}$	788.32	788.33
	$\{[FeL]^{2+}+H^{+}\}^{3+}$	525.88	525.89
	$\{[FeL]^{2+}+2H^{+}\}^{4+}$	394.66	394.67
Fe(II):L5	$\{[L]+H^{+}\}^{+}$	1205.45	1205.45
	$\{[L]+2H^{+}\}^{2+}$	603.23	603.23
	$\{[L]+3H^{+}\}^{3+}$	402.49	402.49
	$\{[L]+4H^{+}\}^{4+}$	302.11	302.12
	$\{[FeL]^{2+}\}$	630.19	630.19
	$\{[FeL]^{2+}+H^{+}\}^{3+}$	420.46	420.46
	$\{[FeL]^{2+}+2H^{+}\}^{4+}$	315.59	315.60
Fe(II):L6	$\{[L]+H^{+}\}^{+}$	1261.50	1261.51

	$\{[L]+2H^+\}^{2+}$	631.25	631.26
	$\{[L]+3H^+\}^{3+}$	421.17	421.17
	$\{[L]+4H^+\}^{4+}$	316.13	316.13
	$\{[FeL]^{2+}\}$	658.21	658.22
	$\{[FeL]^{2+}+H^+\}^{3+}$	439.14	439.15
	$\{[FeL]^{2+}+2H^+\}^{4+}$	329.61	329.61
Zn(II):L1	$\{[L]+H^+\}^+$	882.40	882.40
	$\{[L]+2H^+\}^{2+}$	441.60	441.70
	$\{[L]+3H^+\}^{3+}$	294.72	294.80
	$\{[ZnL]^{2+}\}$	472.62	472.66
Zn(II):L2	$\{[L]+2H^+\}^{2+}$	647.29	647.29
	$\{[L]+3H^+\}^{3+}$	431.86	431.86
	$\{[L]+4H^+\}^{4+}$	324.15	324.15
	$\{[ZnL]^{2+}\}$	678.25	678.25
	$\{[ZnL]^{2+}+H^+\}^{3+}$	425.50	425.50
	$\{[ZnL]^{2+}+2H^+\}^{4+}$	339.63	339.63
Zn(II):L3	$\{[L]+H^+\}^+$	1029.50	1029.49
	$\{[L]+2H^+\}^{2+}$	515.22	515.25
	$\{[ZnL]^{2+}\}$	546.18	546.20
	$\{[ZnL]^{2+}+H^+\}^{3+}$	364.41	364.47
Zn(II):L4	$\{[L]+2H^+\}^{2+}$	761.36	761.37
	$\{[L]+3H^+\}^{3+}$	507.91	507.92
	$\{[L]+4H^+\}^{4+}$	381.67	381.67
	$\{[ZnL]^{2+}\}$	792.32	792.33
	$\{[ZnL]^{2+}+H^+\}^{3+}$	528.55	528.55
	$\{[ZnL]^{2+}+2H^+\}^{4+}$	396.67	396.67
Zn(II):L5	$\{[L]+H^+\}^+$	1205.45	1205.45
	$\{[L]+2H^+\}^{2+}$	603.21	603.23
	$\{[L]+3H^+\}^{3+}$	402.44	402.49
	$\{[ZnL]^{2+}\}$	423.08	423.12
	$\{[ZnL]^{2+}+H^+\}^{3+}$	634.17	634.18
Zn(II):L6	$\{[L]+H^+\}^+$	1261.52	1261.51
	$\{[L]+2H^+\}^{2+}$	631.25	631.26
	$\{[L]+3H^+\}^{3+}$	421.13	421.17
	$\{[ZnL]^{2+}\}$	662.21	662.22
	$\{[ZnL]^{2+}+H^+\}^{3+}$	441.77	441.81

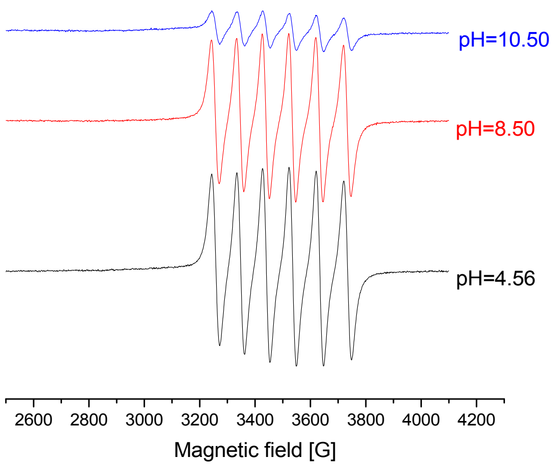

Figure S5 EPR spectrum of the Mn(II)/L1 system, taken in broad pH range at room-temperature (298K).

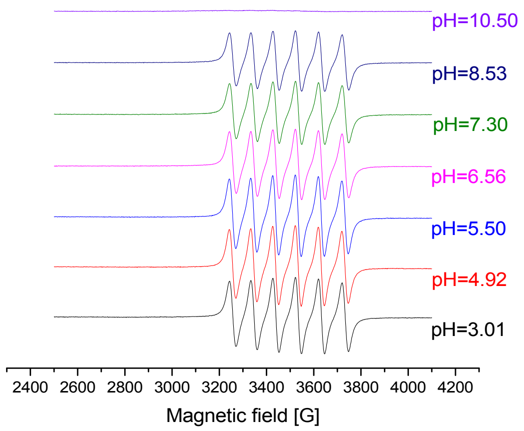

Figure S6. EPR spectrum of the Mn(II)/L2 system, taken in broad pH range at room-temperature (298K).

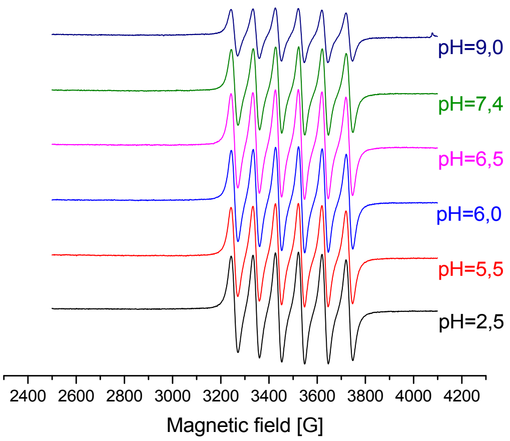

Figure S7. EPR spectra of the Mn(II)/L3 system, taken in broad pH range at room-temperature (298K).

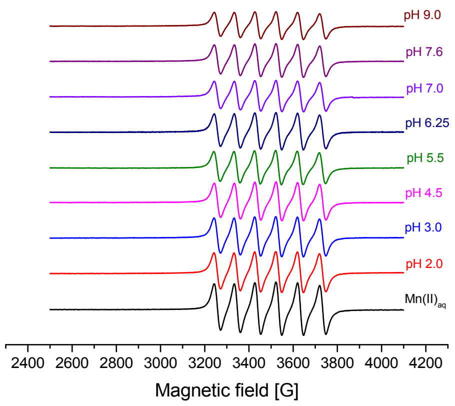

Figure S8. EPR spectra of the Mn(II)/L4 system, taken in broad pH range at room-temperature (298K).

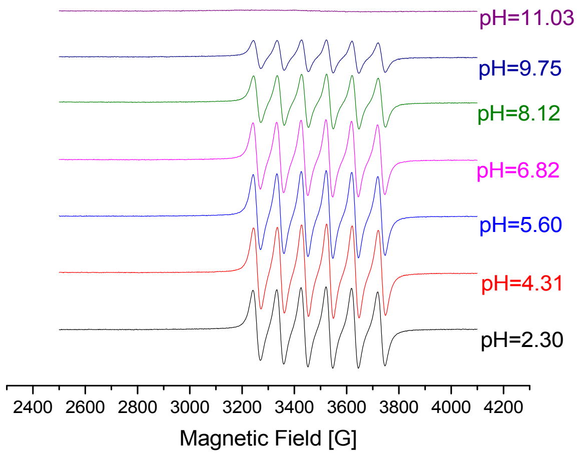

Figure S9. EPR spectra of the Mn(II)/L5 system, taken in broad pH range at room-temperature (298K).

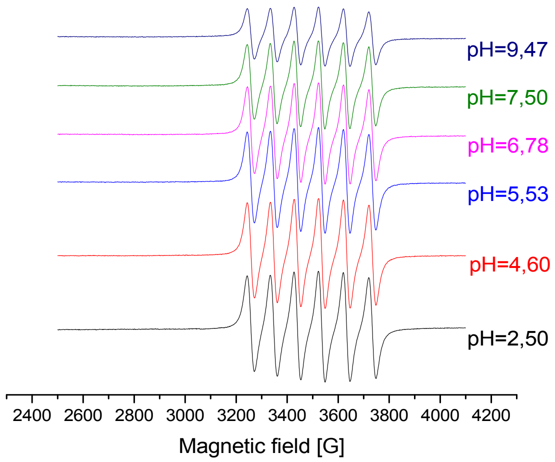

Figure S10. EPR spectra of the Mn(II)/L6 system, taken in broad pH range at room-temperature (298K).

Zn(II)/L1/L2

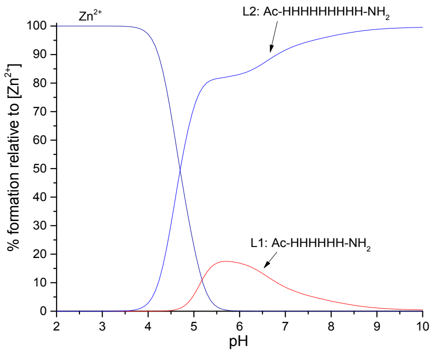

Zn(II)/L2/L3/L4

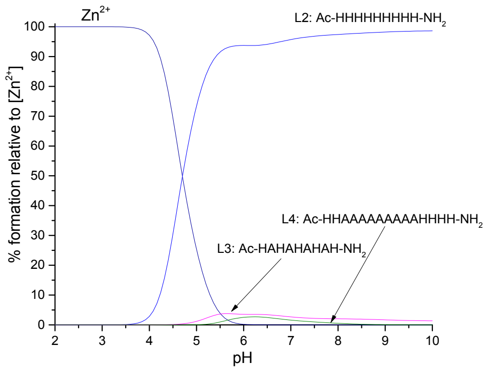

Zn(II)/L2/L3/L5/L6

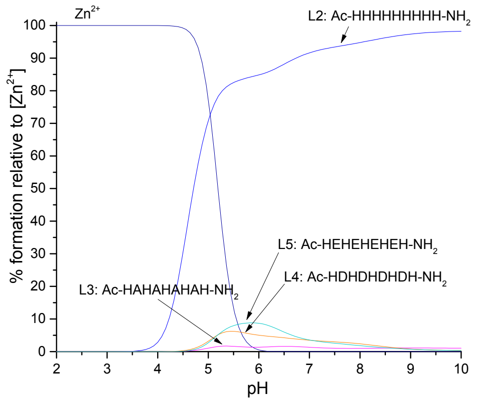

Figure S11. Competition plots between L1/L2; L2/L3/L4 and L2/L3/L5/L6 ligands with Zn(II) ions. The plot describes the hypothetical situation in which equimolar amounts of all reagents are mixed. Conditions: T=298K, *I*=0.1M $NaClO_4$, M:L = 1:1.1; the concentration of all reagents is $1 \cdot 10^{-3}$ M.
